# Astragaloside IV and tumor microenvironmental barriers: immune mechanisms, evidence hierarchy, and implications for checkpoint blockade

**DOI:** 10.3389/fimmu.2026.1991297

**Published:** 2026-10-22

**Authors:** Yingbo Wang, Mingyao Liu, Liping Zhong

**Affiliations:** 1 State Key Laboratory of Targeting Oncology, Guangxi Medical University, National Center for International Research of Bio-targeting Theranostics, Guangxi Key Laboratory of Bio-targeting Theranostics, Collaborative Innovation Center for Targeting Tumor Diagnosis and Therapy, Guangxi Talent Highland of Major New Drugs Innovation and Development, Targeting Theranostics Research Center of Guangxi Higher Education, Guangxi Medical University, Nanning, China; 2 East China Normal University, Shanghai Key Laboratory of Regulatory Biology, Institute of Biomedical Sciences and School of Life Sciences, Shanghai, China; 3 Pharmaceutical College, Guangxi Medical University, Nanning, China

**Keywords:** astragaloside IV, cancer immunotherapy, CD8+ T cells, immune checkpoint blockade, immune exclusion, immunometabolism, tumor microenvironment

## Abstract

Immune checkpoint inhibitors (ICIs) have revolutionized cancer therapy; however, primary and acquired resistance remain prevalent in solid tumors. Challenges such as poor T-cell infiltration, spatial T-cell exclusion, and T-cell dysfunction present distinct yet interrelated obstacles that may hinder checkpoint blockade effectiveness. Astragaloside IV (AS-IV) exhibits anti-inflammatory, antioxidant, endothelial-protective, antifibrotic, and metabolism-modulating properties relevant to the tumor microenvironment (TME). Rather than functioning merely as a conventional systemic immune stimulant, AS-IV may be more effectively viewed as a modulator of local structural, cellular, and metabolic conditions that influence checkpoint-blockade responsiveness. This Review critically examines preclinical evidence linking AS-IV to tumor immune regulation and ICI responses. A total of twenty primary studies comprise the core evidence base: six studies evaluating direct combinations of free AS-IV with checkpoint blockade, nine representative tumor-specific mechanistic investigations without direct ICI testing, and five studies on ICI combinations utilizing AS-IV-containing formulations or delivery systems. Tumor-specific findings connect AS-IV with the PPP1R14B/CX3CL1/CD8^+^ T-cell and MCT1/lactate pathways, macrophage-associated states, cancer-associated fibroblast activity, and context-dependent PD-L1 regulation. Direct combinations of free AS-IV and ICIs have demonstrated superior antitumor activity compared to respective monotherapies in several models, though formal pharmacological synergy remains unproven. Most models lacked adequate characterization of pretreatment spatial immune phenotypes, and increases in CD8^+^ T-cell measures or combination benefits post-treatment do not confirm “cold-to-hot” conversion. Proposed effects on tumor vascular normalization, ECM remodeling, perfusion, and hypoxia primarily rely on indirect or cross-contextual evidence. Thus, AS-IV should be viewed as a potential TME-modulating adjunct to ICIs rather than a well-established component of ICI combination therapy. Further research is necessary to delineate tumor-specific causal mechanisms, pharmacologically relevant exposure levels, responsive TME phenotypes, optimal treatment schedules, and combination safety.

## 1 Introduction

Immune checkpoint inhibitors (ICIs), particularly those targeting programmed cell death protein 1 (PD-1) and programmed death-ligand 1 (PD-L1), have revolutionized cancer treatment, inducing durable responses in a subset of patients (1–3). Nonetheless, substantial primary and acquired resistance is observed in many patients with solid tumors (4, 5). This limited efficacy is attributed to factors beyond immune checkpoint signaling, as effective antitumor immunity also necessitates adequate antigen presentation, T-cell priming and recruitment, tumor infiltration, and sustained effector function (2, 6).

Cold tumors are typically characterized by insufficient infiltration or spatial exclusion of effector T cells, frequently accompanied by impaired T-cell function. In this Review, the cold-tumor framework primarily encompasses poorly infiltrated phenotypes, including immune-desert states, as well as spatially immune-excluded phenotypes; T-cell dysfunction is considered separately as a distinct functional state, while ICI response or resistance is viewed as a treatment outcome. These phenotypes may arise from various abnormalities within the tumor microenvironment (TME), including aberrant vasculature, a dense extracellular matrix (ECM), hypoxia, accumulation of immunosuppressive cells, and metabolic constraints (7). Model classification relies on pretreatment measurements detailed in the primary studies, with operational criteria specified in the Methods section. Consequently, TME normalization has emerged as a complementary strategy to enhance ICI responses by promoting effector T-cell infiltration and supporting antitumor immune function (8–12). This approach presents a pharmacological opportunity to modify local conditions influencing immune-cell access and function, rather than solely depending on stronger systemic immune activation.

Astragaloside IV (AS-IV), a major bioactive saponin extracted from Astragalus, possesses anti-inflammatory, antioxidant, endothelial-protective, and antifibrotic properties. These characteristics align with several determinants of immune exclusion and dysfunction, providing a pharmacological rationale for assessing AS-IV as a TME-modulating adjunct to ICIs. Direct combinations of free AS-IV with PD-1/PD-L1 blockade have been reported in various tumor contexts, including prostate, colorectal, lung, triple-negative breast cancer, and nasopharyngeal carcinoma models (13–18), with several studies noting improved tumor control relative to corresponding monotherapies. However, many of these models lacked adequate characterization of pretreatment spatial immune phenotypes and should not be assumed to represent defined cold or immune-excluded states. Observed combination activity should thus be interpreted independently of claims regarding “cold-to-hot” conversion or pharmacological synergy. Tumor-specific mechanistic studies further link AS-IV to chemokine-associated CD8^+^ T-cell responses, MCT1/lactate-related metabolic suppression, checkpoint regulation, macrophage-associated states, and stromal function. However, the existing tumor-specific and ICI-combination evidence remains preclinical, with considerable variability in mechanistic depth and causal validation across studies.

Previous reviews focusing on AS-IV have examined its anticancer activities, mechanisms across tumor types and signaling pathways, and broader pharmacological properties. Xia et al. discussed AS-IV as a potential anticancer agent (19), Zhou et al. summarized its anticancer effects and mechanisms across tumor contexts (20), and Liang et al. reviewed its wider pharmacological effects (21). Building upon these studies, this Review critically assesses the mechanistic rationale, available evidence, and translational challenges surrounding AS-IV as a candidate TME-modulating adjunct to checkpoint blockade. Distinction is made among direct free AS-IV–ICI combination evidence, tumor-specific mechanistic studies of free AS-IV, AS-IV-containing formulation or delivery studies, and pharmacological extrapolations from non-tumor models. Rather than cataloging the diverse pharmacological effects of AS-IV, the focus lies on how AS-IV-associated mechanisms may modify immune-cell access, suppressive immune states, T-cell functional competence, immunometabolic stress, and the local context influencing checkpoint blockade effectiveness. This perspective, emphasizing evidence stratification, specifically targets checkpoint-blockade relevance while distinguishing tumor-specific evidence from broader pharmacological plausibility, and considers pretreatment phenotypes, causal support, and exposure feasibility.

## 2 Methods: literature scope and evidence categorization

A structured literature search was conducted in PubMed/MEDLINE and Web of Science Core Collection from database inception to 6 October 2026. The primary search combined “Astragaloside IV” or “Astragaloside-IV” with cancer- and tumor-related terms using database-specific syntax and fields. Reference lists of relevant primary studies and recent reviews were also screened. Additional supporting evidence regarding non-tumor pharmacology, pharmacokinetics, safety, and cross-context mechanistic plausibility was identified through targeted searches and citation tracking. A separate search of ClinicalTrials.gov, the WHO International Clinical Trials Registry Platform (ICTRP), and the Chinese Clinical Trial Registry (ChiCTR) was conducted through 7 October 2026 to identify registered human oncology studies evaluating AS-IV itself as an intervention, either alone or in combination with another anticancer treatment, including immune checkpoint blockade. The PubMed/MEDLINE and Web of Science searches yielded 469 records; after removing 128 cross-database duplicates, 341 unique records remained. Full database and trial-registry search strategies, along with the operational definitions used in this Review, are detailed in **Supplementary Table 1**.

Evidence was organized into four categories: (i) direct combinations of free AS-IV with PD-1/PD-L1 blockade (Category A); (ii) representative tumor-specific mechanistic studies of free AS-IV without direct checkpoint-blockade testing (Category B); (iii) ICI-combination studies using AS-IV-containing formulations or delivery systems (Category C); and (iv) cross-context pharmacological evidence supporting mechanistic plausibility. Categories A–C comprised the principal primary-study evidence and were mutually exclusive for study counting. Categories A and C included identified studies directly testing ICI-based combinations, while Category B was defined as a representative, non-redundant mechanistic stratum rather than an exhaustive catalog of tumor-related AS-IV studies. Distinct checkpoint-relevant mechanisms involving T-cell responses, checkpoint regulation, stromal or macrophage-associated states, and immunometabolic constraints received priority. Closely overlapping mechanistic studies were treated as corroborative evidence rather than counted separately. The principal evidence framework encompassed 20 studies: six in Category A, nine in Category B, and five in Category C.

Assessment of pretreatment immune phenotype occurred separately from treatment response. Immune-desert status required evidence of low or absent pretreatment T-cell abundance, while immune-excluded status necessitated spatial evidence of T-cell retention in stromal or marginal regions with limited penetration into tumor parenchyma. Tumor type, individual markers, post-treatment CD8^+^ T-cell increases, or combination efficacy alone were not utilized to infer a cold-tumor phenotype. T-cell dysfunction, exhaustion, and ICI response or resistance were also evaluated separately. CD8^+^ T-cell counts, densities, proportions, and staining signals were not used interchangeably, and the terms “recruitment”, “infiltration,” and “functional improvement” were used only when supported by corresponding migration or trafficking, tumor-tissue localization, or functional evidence. Detailed criteria are presented in **Supplementary Table 1**.

For each principal study, considerations included model and control design, AS-IV regimen and purity (where reported), sample size, randomization and blinding, statistical analysis, immune assays, mechanistic perturbation, and exposure information. Missing information was recorded as not reported or unclear rather than inferred. Direct target claims necessitated evidence of compound–target interaction beyond pathway association, while “pharmacological synergy” was reserved for studies employing a defined synergy model or formal interaction analysis. Otherwise, combination effects were described in relation to corresponding monotherapies. **Table 1** summarizes the principal evidence, with a detailed methodological appraisal provided in **Supplementary Table 2**. This Review serves as a critical narrative assessment rather than performing a formal meta-analysis or producing an aggregate quality score.

**Table 1:** Principal study-level evidence for AS-IV-related tumor immune mechanisms and ICI combination strategies.

A. Direct combinations of free AS-IV with ICIs (n = 6)						
Study and model	AS-IV regimen and purity	ICI/comparator design	Key antitumor and immune findings/principal statistics	Pretreatment immune phenotype/spatial CD8 evidence	Mechanistic evidence	Evidence interpretation/major limitations
Yang et al., 2026 (13); subcutaneous prostate cancer in RM1/C57BL/6J mice and tumor cells–CD8 co-culture; *in vivo*, n = 5 per group	>98% (HPLC); 40 mg/kg, administered by oral gavage, every 3 days	Anti-PD-1 regimen + anti-PD-1 ± AS-IV comparison + AS-IV monotherapy was evaluated separately	Greater tumor control was reported with anti-PD-1 plus AS-IV than with anti-PD-1 alone; CX3CL1 expression and CD8 staining in tumors increased, and CD8^+^ T-cell chemotactic activity increased *in vitro*	Pretreatment phenotype not established; post-treatment CD8 staining was reported without quantitative spatial compartment analysis	SPR with recombinant PPP1R14B supported AS-IV–PPP1R14B binding; cellular CETSA was consistent with target engagement; PPP1R14B overexpression, knockdown and downstream interventions; protein-stability/ubiquitination, chemotaxis and co-culture assays	AS-IV monotherapy and ICI combination treatment were assessed in separate experiments; no matched four-arm comparison was reported. The *in vivo* necessity of the PPP1R14B/CX3CL1/CD8^+^ T-cell axis for the additional combination benefit has not been established
Liu et al., 2020 (14); Syngeneic CT26 tumors in BALB/c mice and bone marrow-derived macrophages; efficacy: n = 8 per group; immunological analysis: n = 5 per group	Purity: NR; 15 mg/kg, every 3 days, for a total of 3 doses; inconsistent records regarding route of administration	Anti-PD-1 (BE0146) 2.5 mg/kg, every 3 days, for a total of 3 doses; inconsistent records regarding the route of administration; four groups were established: control, AS-IV, anti-PD-1 and combination therapy	Greater tumor control was reported in the combination group than with either monotherapy; the proportion of CD8^+^ T cells among CD45^+^ cells in tumor tissue increased; and changes were observed in macrophage markers and the cytokine profile	Pretreatment phenotype not established; post-treatment tumor flow cytometry was reported without spatial CD8 compartment analysis	Analysis of the phenotype, transcriptome and proteome of bone marrow-derived macrophages; flow cytometric analysis of CD206/MHC-II expression in tumor CD11b^+^F4/80^+^ cells	Macrophage depletion or adoptive transfer was not reported to establish a causal contribution to the combination benefit.
Wu et al., 2024 (15); LLC/C57BL/6J subcutaneous lung cancer; sample size for animal group: NR	Purity: NR; 40 mg/kg, every 3 days; route of administration not specified	Anti-PD-1–10 mg/kg, administered intraperitoneally twice weekly; four groups were established: control, AS-IV, anti-PD-1 and combination therapy	Tumor volume and weight were lower with the combination than with either monotherapy. Tumor CD86 immunofluorescence signals increased and CD206 signals decreased; CD3 and CD69 immunofluorescence signals increased in both tumors and spleen	Pretreatment phenotype not established; no CD8 or spatial T-cell compartment analysis was reported	Immunofluorescence staining; detection of PI3K/Akt and ERK-related proteins	Based primarily on the tissue immunofluorescence signals; no reports on co-labeling of the macrophage lineage or CD8 cytotoxic function assays
Wei et al., 2026 (16); 4T1/BALB/c subcutaneous breast cancer and 4T1 and MDA-MB-231 cells; a total of 12 animals *in vivo*, divided into 4 groups	Purity: NR; 50 mg/kg, administered by oral gavage, daily; concentration for the primary mechanism *in vitro*: 80 μg/mL	Anti-PD-L1–5 mg/kg, administered intraperitoneally every 3 days; four groups were established: control, AS-IV, anti-PD-L1 and combination therapy	Greater tumor control was reported with the combination than with either monotherapy. Flow cytometry of tumor-derived cell suspensions showed higher reported percentages of CD3^+^CD8^+^, CD8^+^IFN-γ^+^, and CD8^+^granzyme B^+^ cells (parent populations insufficiently specified). STING/TBK1-related signaling and total PD-L1 protein increased in tumor cells *in vitro*	Pretreatment phenotype not established; post-treatment tumor flow cytometry was reported without spatial CD8 compartment analysis	The H-151 intervention supports STING involvement in *in vitro*-related changes	The total sample size *in vivo* was small (12 animals divided into 4 groups); no reports were provided on the direct binding of AS-IV to cGAS or STING or on the validation of their necessity *in vivo*; at 80 μg/mL, there was a marked decrease in cell viability
Zeng et al., 2026 (17); C666-1/huHSC-NCG immunohumanized mice and nasopharyngeal carcinoma cells–CD8 co-culture; n = 9 per group	Purity: NR; *in vivo*: 40 mg/kg daily, route of administration not specified; *in vitro*: 100–400 μM	*In vitro*: atezolizumab 5 μg/mL. *In vivo*: four-group checkpoint-blockade experiment; antibody identity and dose were inconsistently reported (see **Supplementary Table 2**)	Tumor growth was inhibited; the proportion of CD8^+^ cells in tumor tissue, as determined by flow cytometry, was increased (parent population not specified); in co-culture, LDH release, IFN-γ levels in the supernatant, and perforin and granzyme B levels in the collected CD8^+^ T cells were increased; SATB2/Wnt/β-catenin-related signaling, as well as PD-L1 mRNA and total protein levels, were decreased	Pretreatment phenotype not established; post-treatment CD8 assessment was reported without quantitative spatial compartment analysis	SATB2 knockdown and overexpression; Wnt pathway manipulation and co-culture functional assays	The *in vivo* ICI regimen is unclear, and no direct binding between AS-IV and SATB2 has been reported
Meng et al., 2021 (18); subcutaneous Lewis lung carcinoma in C57 mice; mechanistic studies in lung cancer cells	*In vivo*: 5 mg/kg by injection; specific route and schedule unclear. *In vitro*: 5–10 μM. Purity: NR	Anti-PD-1 (Bio X Cell), 200 μg/mouse, i.p.; clone and schedule: NR. Control, AS-IV, anti-PD-1 and combination groups	Greater tumor-growth inhibition with combination treatment; increased reported CD3^+^CD8^+^ T-cell proportions in tumor-derived lymphocyte preparations	Pretreatment phenotype not established; no spatial CD8 assessment was reported	Co-IP/pull-down and truncation studies supported GBP1–IDO1 interaction. AS-IV reduced co-IP association and medium IDO1, while intracellular IDO1 increased	Combination-group n and flow-cytometry denominator unclear; no formal synergy or direct T-cell function/exhaustion assessment; necessity of the proposed mechanism for combination benefit not established

B. Representative tumor-specific mechanistic studies of free AS-IV without direct ICI combination testing (n = 9)						
Study and model	AS-IV regimen and purity	ICI/comparator design	Key antitumor and immune findings/principal statistics	Pretreatment immune phenotype/spatial CD8 evidence	Mechanistic evidence	Evidence interpretation/major limitations
Zhao et al., 2026 (75); subcutaneous oral squamous cell carcinoma (SCC7/C3H) and co-culture with CAL27 and SCC25–CD8; a total of 12 animals *in vivo*, divided into 2 groups	>98%; 50 mg/kg, intraperitoneal injection, daily; inconsistent *in vitro* concentration data	ICI not tested; MCT1 overexpression and lactate-related experiments were performed	Tumor growth was inhibited; lactate levels, acidification and tumor-cell surface PD-L1 decreased in co-culture; LDH release and IFN-γ, TNF-α, granzyme B and perforin levels in the co-culture supernatant increased; tumor CD8 staining increased	The pretreatment spatial phenotype had not been established; CD8 tissue staining and multiplex immunohistochemistry were performed, but no quantitative CD8 tissue compartmentalization was reported	MST and SPR detected AS-IV–MCT1 interactions; photoaffinity pull-down in cell lysates provided additional interaction evidence; MCT1 overexpression and mRNA-stability experiments further implicated the MCT1/lactate axis	Tumor-specific mechanistic evidence supports the MCT1/lactate axis; checkpoint blockade was not tested, so this is not direct evidence of additional benefit from combining AS-IV with checkpoint blockade. Target engagement in intact cells, direct MCT1 transport inhibition and *in vivo* MCT1 dependence remain unestablished
Ma et al., 2023 (80); co-culture of Huh-7 and SMMC-7721–PBMC cells, and subcutaneous HCC in Huh-7/BALB/c nude mice; n = 5 per group	Purity: NR; *in vitro*: primarily 80 μg/mL;*in vivo*: 50–150 mg/kg, administered by oral gavage daily	ICI not tested; comparison of interventions affecting IFN-γ induction and the expression of miR-135b-5p and CNDP1	AS-IV reduced IFN-γ-induced tumor-cell surface PD-L1 and increased tumor-cell killing in PBMC co-culture; tumor growth was suppressed in nude mice	Pretreatment phenotype not established; no spatial tumor CD8 assessment was reported	miRNA mimics, CNDP1 expression modulation and dual-luciferase reporter assays	The tumor-suppressing results in nude mice do not establish a mechanism dependent on mature T cells; there is a record of misidentification for SMMC-7721; no direct AS-IV–target interaction was demonstrated
Xu et al., 2018 and 2023 correction (62, 63); THP-1 macrophage–lung cancer cell experiments and the LLC/C57BL/6J model; *in vivo* immunological analyses: n = 6–7 per group	>98%; 80 μM *in vitro*; refer to the corrected figure; 40 mg/kg *in vivo*, administered by oral gavage, daily	ICI not tested; includes macrophage induction, conditioned medium and AMPK knockdown controls	AS-IV reduced M2-associated markers, and conditioned medium from treated macrophages was less able to promote tumor-cell migration and invasion. The measured M1-associated markers did not increase substantially. Tumor growth and lung metastasis decreased, without significant changes in CD3^+^, CD4^+^ or CD8^+^ T-cell proportions	The pretreatment spatial phenotype had not been established; no evidence of CD8 tissue compartmentalization was reported	AMPK knockdown; conditioned medium and migration and invasion assays	Supports modulation of selected macrophage-associated states rather than complete M2-to-M1 conversion; *in vivo* TAM causality was not established. Formal correction (63) incorporated
Zhang et al., 2014 (68); IDO-overexpressing 3LL-Luc/C57BL/6 *in situ* lung cancer; n = 10 per group	Purity: NR; 40 mg/kg, administered orally, daily	ICI not tested; comparison groups included saline, 1-MT and paclitaxel	Slowed tumor progression and prolonged survival; a decrease in the proportion of CD4^+^CD25^+^ cells and an increase in the proportion of CD8^+^CD28^+^ cells in the spleen, accompanied by reduced IDO expression	Pretreatment phenotype not established; immune subsets were assessed predominantly in spleen, with no spatial tumor CD8 assessment	IDO overexpression background, 1-MT comparison and detection of immune subsets	Tumor-infiltrating Tregs or CD8^+^ cytotoxic function were not directly assessed; no data were reported on IDO binding, enzymatic activity or kynurenine measurements
Wang et al., 2017 (50); the effects of GCAF derived from a single patient and its conditioned medium on BGC-823 cells	Purity: NR; GCAF treated with 10–40 μM for 72 hours	ICI not tested; includes normal fibroblasts, GCAF, solvent and miRNA intervention controls	In GCAF, M-CSF is downregulated and TIMP2 is upregulated; the role of conditioned medium in promoting tumor cell proliferation, migration and invasion is attenuated	NA; no intact-tumor spatial immune phenotype was assessed	miR-214 inhibition and miR-301a overexpression using a mimic; functional studies using conditioned medium	CAF derived from a single patient; BGC-823 has a record of misidentification; *in vivo* ECM, tissue mechanics and spatial T-cell exclusion were not evaluated
Liu et al., 2023 (51); primary gastric CAFs co-cultured with BGC-823/MGC803 cells; n = 3 replicates reported; donor number NR	≥98% (HPLC); 10–40 μM for 72 h; key functional assays used 40 μM	ICI not tested; normal fibroblasts, vehicle, shRNA and empty-vector controls	Reduced CAF HOXA6/ZBTB12 expression and tumor-cell growth, migration and invasion in co-culture	NA; *in vitro* study without immune-cell or spatial CD8 assessment	HOXA6/ZBTB12 knockdown and overexpression; promoter reporter and ChIP assays	Donor replication unclear; problematic tumor-cell identities; no *in vivo* or immune-causality validation
Liang et al., 2023 (73); AOM/DSS colitis-associated tumorigenesis model and LPS-stimulated IEC-6 cells; primary *in vivo* outcome: n = 6 per group	≥98% (HPLC); 20, 40 and 80 mg/kg, administered by oral gavage, daily	ICI not tested; included control, model, mesalazine and AS-IV + GW9662 comparison groups, etc	A reduction in the number of colon tumors, inflammation and oxidative DNA damage was reported, together with changes in PPARγ/Nrf2/HO-1-related signaling	Inflammation-related tumorigenesis model; the pretreatment immune phenotype of established tumors has not been characterized, and no spatial assessment of CD8^+^ cells has been reported	SPR; GW9662 intervention *in vivo* and *in vitro*	Provides PPARγ-related target-interaction and pharmacological evidence in inflammation-associated tumorigenesis, but does not establish T-cell immune regulation in established tumors or ICI responsiveness
Min et al., 2022 (64); Huh-7/THP-1-derived macrophage co-culture and subcutaneous Huh-7/M2-macrophage co-implantation in BALB/c nude mice; n = 10 per group reported	Purity NR; 120 μM for mechanistic experiments; 20, 40, 80 or 100 mg/kg by oral gavage every 3 days, starting when tumors reached approximately 5 mm	ICI not tested; untreated/vehicle controls, macrophage induction with or without AS-IV, and TLR4 siRNA conditions	Reduced Huh-7 proliferation, migration and invasion; reduced macrophage CD206 positivity and CD209 and TGF-β expression *in vitro*. Tumor volume and weight decreased at 40–100 mg/kg	Pretreatment spatial phenotype not established; CD8 infiltration and tissue compartmentalization not reported	TLR4 siRNA perturbation and pathway immunoblotting; combined TLR4 knockdown and AS-IV further reduced M2-associated markers and TLR4/NF-κB/STAT3 pathway readouts	Immunodeficient host; direct effects on tumor-cell viability complicate macrophage-specific attribution; *in vivo* macrophage dependence not established; inconsistent descriptions of siRNA-treated cells and SD/SEM reporting
Zhou et al., 2024 (65); MC38/RAW264.7 conditioned-medium experiments and an intrasplenic MC38 liver metastasis model in C57BL/6J mice; n = 3 per group *in vivo*	Purity NR; 12.5–50 μM in key *in vitro* experiments; 50 mg/kg by oral gavage daily for 5 weeks, starting 1 week before tumor inoculation	ICI not tested; PBS/model, MC38-EV and GW4869 comparator groups; untreated and conditioned-medium controls *in vitro*	Reduced tumor-cell EV release and macrophage-mediated CRC-cell migration; reduced liver metastatic burden, with changes in macrophage-associated phenotypes	Pretreatment spatial phenotype not established; CD8 infiltration and tissue compartmentalization not reported	EV characterization and quantification; conditioned-medium experiments and GW4869 comparison; reduced nSMase2/Rab27a expression; EV add-back rescue reported in supplementary experiments	Small animal groups and dosing before tumor inoculation limit therapeutic inference; direct molecular targets and *in vivo* dependence on EV suppression or macrophage changes not established

C. ICI combination studies using AS-IV-containing formulations or delivery systems (n = 5)						
Study and model	AS-IV regimen and purity	ICI/comparator design	Key antitumor and immune findings/principal statistics	Pretreatment immune phenotype/spatial CD8 evidence	Mechanistic evidence	Evidence interpretation/major limitations
Guo et al., 2024 (58); H22-Luc/C57BL/6 *in situ* HCC; end-point tumor analysis n = 5 per group, survival n = 8 per group	AS-IV, purity NR; TSA/AS-IV co-loaded magnetic MOF platform, 15 mg/kg platform mass i.v. daily; AS-IV-equivalent dose NR	Anti-PD-1–5 mg/kg, administered intravenously every 4 days; groups included PBS, free TSA/AS-IV, blank carrier, Hm@TSA/As-MOF, anti-PD-1, and Hm@TSA/As-MOF plus anti-PD-1	Greater tumor control was reported with the combination; T-bet^+^ and Eomes^+^ proportions among CD8^+^ TILs increased; co-staining signals for CD8 and IFN-γ or granzyme B increased in tumor tissue; changes were reported in platform-related indicators of vascular maturity, perfusion, leakage and hypoxia	The pretreatment spatial phenotype had not been established; TILs were detected, but no quantitative data on CD8 tissue compartmentalization were reported	Vascular function, hypoxia, TIL phenotype and effect assessment; delivery evaluation	Lack of single-component and single-component vehicle controls; tracer distribution does not correspond to actual AS-IV pharmacokinetics. The reported H22-Luc/C57BL/6 pairing lacks documentation of tumor–host strain compatibility, which should be considered when interpreting the TIL and anti-PD-1 findings
Wei et al., 2025 (77); 4T1-Luc/BALB/c *in situ* breast cancer; tumor response: n = 6 per group; survival: n = 8 per group	AS-IV purity >98%; intravenous administration via co-loaded liposomes, AS-IV equivalent 1.5 mg/kg, oxymatrine 2.0 mg/kg	Anti-PD-1–5 mg/kg, administered intravenously every 4 days; groups: control, free oxymatrine + AS-IV combination, anti-PD-1, oxymatrine/AS-IV co-loaded liposomes, and co-loaded liposomes plus anti-PD-1	Greater tumor control and longer survival were reported with the combination; CAF-related markers and collagen deposition decreased; basal and maximal oxygen consumption rates of isolated TILs increased; IFN-γ^+^ and granzyme B^+^ proportions among CD8^+^ TILs increased	The pretreatment spatial phenotype had not been established; stroma and TIL analyses were performed, but no quantitative CD8 tissue compartmentalization was reported	CAF and TIL respiration and CD8 effector cell assays; AS-IV pharmacokinetic measurements	Provides quantitative formulation-related AS-IV exposure data, but the independent contributions of AS-IV, oxymatrine, and the delivery system cannot be separated
Liu et al., 2025 (72); a pulmonary tumor model in LLC-Luc/C57BL/6J mice via tail vein inoculation; the combined experimental methods documented a total of 12 mice divided into 4 groups	Purity: NR; AS-IV/polyphyllin VII co-loaded liposomes, administered by nebulizer inhalation, daily; the doses reported in the study were 5 and 4 mg/kg respectively	Anti-PD-1 (Leinco, P504) 10 mg/kg, administered intraperitoneally every 3 days; four groups were established: PBS, anti-PD-1, inhaled formulation and combination therapy	The combination therapy resulted in a reduction in pulmonary tumor nodules compared with the two treatments administered separately; the proportion of PD-1/TIM-3 co-positive cells among CD8^+^ T cells in lung tissue decreased, while the proportions of CD4^+^Foxp3^+^ and CD8^+^ granzyme B^+^ cells among CD3^+^ cells decreased and increased, respectively	The pretreatment spatial phenotype had not been established; although tissue staining and flow cytometry were performed, no quantitative data on CD8 tissue distribution were reported	Tissue transcriptomics; detection of p-STAT5, BLIMP1 and related transcripts; comparison including IL-2 inhibitors; insufficient reporting of key experimental conditions	Lack of a single-component control; failure to establish acquired ICI resistance and the necessity of the pathway for the benefit of combination therapy; 24-hour drug concentrations in lung tissue were measured by HPLC, but no complete PK curve was provided; see table notes for discrepancies in modeling and exposure reporting
Guo et al., 2023 (78); orthotopic H22-Luc/C57BL/6 HCC; efficacy/survival: n = 8 per group; key flow assays: n = 5; Seahorse assays: n = 3	AS-IV purity: NR; PmMN@Om&As, 15 mg/kg (platform mass), i.v. daily with magnetic targeting. Free control: oxymatrine 2 mg/kg + AS-IV 1.5 mg/kg	Anti-PD-1 (Bio X Cell; clone/catalog NR), 5 mg/kg i.v. every 4 days. Groups: PBS, free oxymatrine + AS-IV, PmMN, anti-PD-1, PmMN@Om&As, and PmMN@Om&As plus anti-PD-1	Greater tumor control and longer survival with the combination; α-SMA, collagen, FAP and CXCL12 measures decreased; oxygen consumption in isolated TILs increased; IFN-γ^+^ and granzyme B^+^ proportions among CD8^+^ TILs increased	Pretreatment spatial phenotype not established; post-treatment flow cytometry and CD8 staining without tumor-nest, stromal or margin quantification	Separate oxymatrine/AS-IV comparisons in TGF-β1-treated NIH3T3 and CTLL-2/H22 models; carrier controls, Seahorse assays and mitochondrial phenotyping	No matched *in vivo* single-drug or single-drug-loaded controls; carrier also altered TIL metabolism; no AS-IV PK or immune-causality validation. H22-Luc/C57BL/6 strain compatibility requires clarification
Kong et al., 2024 (79); orthotopic MB-49/C57BL/6 bladder cancer; efficacy/survival: n = 5 per group; IHC/flow: n = 3	Purity: NR. AS-IV-loaded micelles; tail-vein injection on days 5, 8, 11 and 14 (10 mL/kg). Reported AS-IV amount: 58.44 μg; dose normalization unclear	Anti-PD-L1 (Bio X Cell, BE0101), reported amount 318 μg; normalization unclear. Groups: PBS, PP, PPA, PPA + aPD-L1 and PPA@aPD-L1; free antibody given 8 h after PPA	PPA@aPD-L1 reduced tumor volume and showed longer reported survival; TGI: 84.6% versus PBS. CD8 IHC signal and reported CD3^+^CD8^+^ proportion increased; Treg proportion decreased; tumor IFN-γ increased and IL-10 decreased	Pretreatment phenotype not established; post-treatment CD8 IHC was reported without separate tumor-nest, stromal, or margin quantification	Fluorescent carrier tracing; reduced p-STAT3/p-p65 and nuclear localization in tumor cells; reduced tumor p-STAT3/p-p65. Pathway necessity not established	No antibody-only or antibody-decorated empty-micelle controls; conjugation and dosing sequence differed together. Flow-cytometry reporting requires clarification (see notes)

1. Categories A–C denote evidence type rather than methodological quality. Category B is a representative, non-redundant mechanistic stratum rather than an exhaustive catalog of tumor-related AS-IV studies. “Pretreatment phenotype not established” indicates that available baseline data were insufficient to classify a model as immune-desert or immune-excluded; post-treatment cell counts, proportions, or staining were not used to infer pretreatment phenotype.

2. No ICI-combination study included in this table reported formal pharmacological synergy analysis using a defined reference model. Greater activity than the corresponding monotherapies was therefore interpreted as combination activity rather than pharmacological synergy. Quantitative AS-IV pharmacokinetic data were available in study (77); carrier tracing or single-time-point tissue concentrations were not considered equivalent to full pharmacokinetic assessment.

3. NR, not reported in the materials examined; NA, not applicable. AS-IV purity, dose, route, sample size, and statistical information were recorded as reported and were not inferred when unclear. Detailed appraisal of randomization, blinding, statistical methods, flow-cytometry reporting, cell-line identity, corrections, and study-specific reporting discrepancies is provided in **Supplementary Table 2**.

NR, not reported in the materials examined; NA, not applicable; ICI, immune checkpoint inhibitor; GCAF, gastric cancer-associated fibroblast; ECM, extracellular matrix; TIL, tumor-infiltrating lymphocyte; PBMC, peripheral blood mononuclear cell; HCC, hepatocellular carcinoma; SPR, surface plasmon resonance; MST, microscale thermophoresis; CETSA, cellular thermal shift assay; PK, pharmacokinetics; TSA, tanshinone IIA; MOF, metal–organic framework; IHC, immunohistochemistry; TGI, tumor growth inhibition; EV, extracellular vesicle; CRC, colorectal cancer; SD, standard deviation; SEM, standard error of the mean. For study (79): PP, empty micelles; PPA, AS-IV-loaded micelles; PPA + aPD-L1, PPA followed 8 h later by free anti-PD-L1; PPA@aPD-L1, AS-IV-loaded micelles bearing surface-conjugated anti-PD-L1.

## 3 Mechanisms limiting immune infiltration and T-cell function in the tumor microenvironment

Effective immune checkpoint blockade necessitates a functional cancer-immunity cycle, which includes key processes such as antigen presentation, T-cell activation, T-cell infiltration into tumors, and effector-cell-mediated killing (6). These processes may be constrained at multiple interconnected levels: abnormal vasculature and stroma can hinder immune-cell access, suppressive immune-cell states may limit local effector activity, and metabolic stress can negatively impact T-cell fitness and function (7). Collectively, these structural, cellular, and metabolic constraints form a conceptual framework for interpreting the AS-IV-related evidence discussed below, while the immune phenotype and ICI response of individual models are evaluated independently based on reported evidence in each primary study (**Figure 1**).

**Figure 1:**
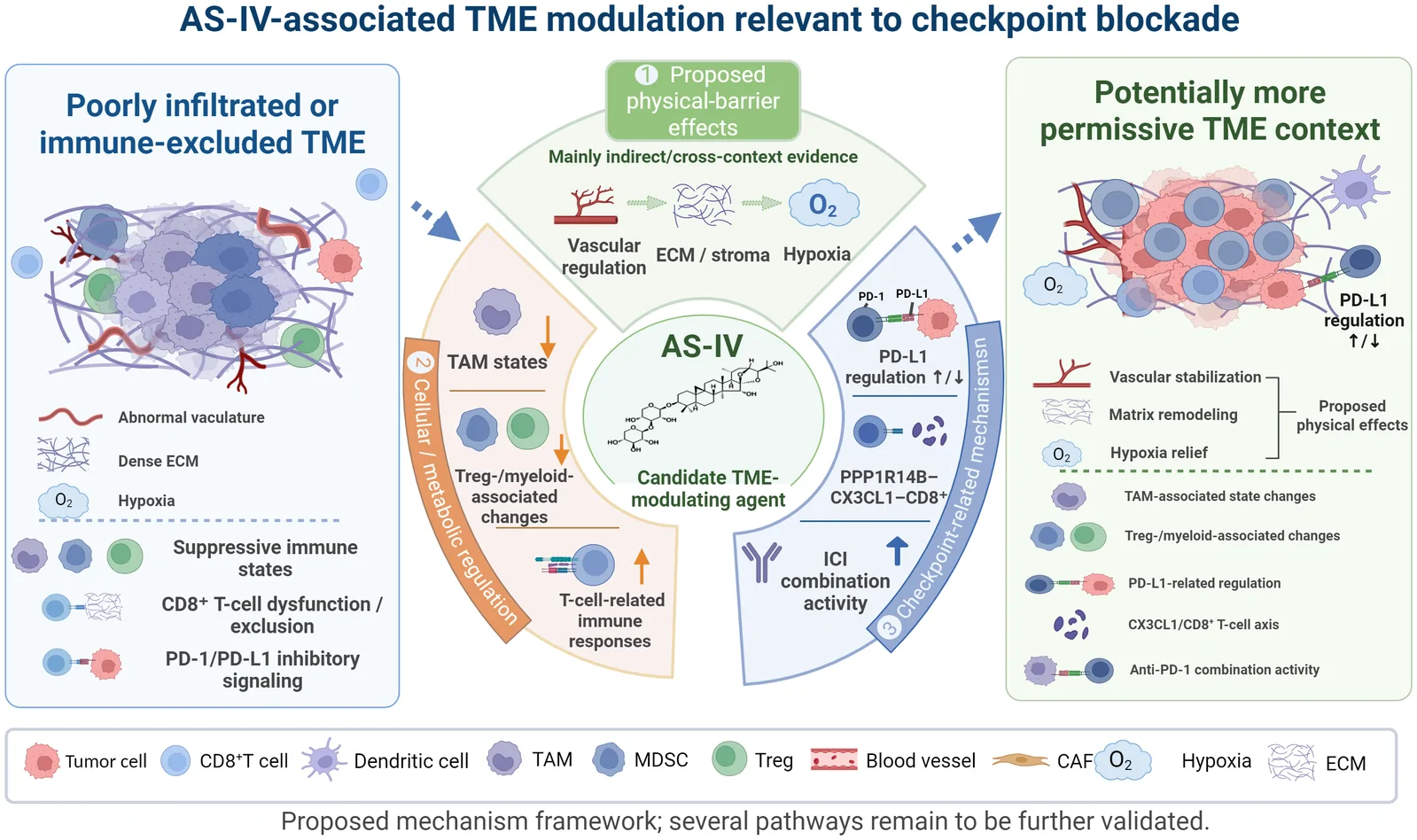
Conceptual framework for AS-IV-associated modulation of tumor microenvironment features relevant to checkpoint blockade. Poor T-cell infiltration, spatial immune exclusion, suppressive immune-cell states, T-cell dysfunction, abnormal vasculature, dense extracellular matrix (ECM), and hypoxia are shown as distinct but interacting barriers to antitumor immunity. Tumor-specific studies link AS-IV to changes in macrophage-associated states, T-cell-related responses, context-dependent PD-L1 regulation, and the PPP1R14B/CX3CL1/CD8+ T-cell axis. Proposed effects on vascular regulation, ECM or stromal remodeling, perfusion, and hypoxia are supported mainly by cross-context or formulation-based evidence and remain to be established for free AS-IV in tumors. Direct studies of free AS-IV combined with PD-1/PD-L1 blockade support additional combination activity, but most did not characterize pretreatment spatial immune phenotypes or formally assess pharmacological synergy. The right-hand panel therefore depicts a potentially more permissive TME context rather than demonstrated “cold-to-hot” conversion. Created in https://BioRender.com.

### 3.1 Physical barriers associated with abnormal vasculature, dense stroma, and hypoxia

Sustained proangiogenic signaling can lead to disorganized tumor vasculature characterized by abnormal permeability and poor perfusion (8, 9, 22). These abnormalities can impair oxygen and drug delivery and restrict effector T-cell movement across the endothelium into tumor parenchyma (23, 24). Additionally, poor perfusion may promote hypoxia and HIF-related signaling, further contributing to immune suppression and metabolic adaptation (25–28). The deposition and cross-linking of the ECM by cancer-associated fibroblasts (CAFs) can increase tissue stiffness and impede cellular movement (29). Solid stress from tumor growth and matrix accumulation can also compress microvessels (10, 12, 30). In metastatic urothelial carcinoma, fibroblast-associated TGF-β signaling has been linked to CD8^+^ T-cell exclusion and reduced response to PD-L1 blockade. In related mouse models, combined TGF-β and PD-L1 blockade has enhanced T-cell infiltration and antitumor activity (31). Collectively, vascular dysfunction, stromal remodeling, and hypoxia create interconnected physical constraints that limit immune-cell infiltration and contribute to ICI resistance.

### 3.2 Functional constraints mediated by suppressive immune-cell networks

In addition to physical barriers to immune-cell access, tumor-associated macrophages (TAMs), myeloid-derived suppressor cells (MDSCs), and regulatory T cells (Tregs) further constrain local antitumor immunity. The effects of these cells depend on their functional state and the local tissue environment, rather than on abundance alone. TAMs exhibit substantial heterogeneity, and their functional states can affect T-cell recruitment and effector activity. A simplistic M1/M2 classification fails to adequately capture this variation (32–34). MDSCs can suppress T-cell responses through mechanisms such as nutrient depletion and oxidative stress (35–37). FOXP3^+^ Tregs can inhibit effector T-cell proliferation and cytotoxic activity via CTLA-4-related mechanisms and cytokines like IL-10 and TGF-β (38). Moreover, these populations interact with their surrounding microenvironment: hypoxia and inflammation may enhance MDSC accumulation (37), while stromal–myeloid interactions can reinforce immune suppression and T-cell exclusion (12, 33, 34). Thus, structural and cellular barriers are interdependent, and their effects are further influenced by the metabolic conditions within the TME.

### 3.3 Metabolic constraints and T-cell dysfunction

Metabolic competition can further restrict antitumor immunity. Tumor metabolic reprogramming modifies local nutrient availability, while lactate accumulation, acidification, and hypoxia contribute to metabolic stress (39). Glucose consumption by tumor cells can inhibit glycolysis, mTOR signaling, and IFN-γ production in effector T cells (40). Evidence related to the Warburg effect and LDHA-dependent lactate production further links tumor lactate accumulation to extracellular acidification, myeloid suppression, and impaired T-cell activity, providing a mechanistic foundation for considering lactate-related metabolic constraints within the TME (41, 42).

The immune effects of lactate are context-dependent. While lactate may contribute to immune suppression, it can also, under certain conditions, support T-cell metabolism and function. The effects of protein lactylation similarly depend on cell type and state (43). Activated T cells require sufficient metabolic support for proliferation and cytotoxic activity (44). Since tumor cells and effector T cells share some metabolic requirements, broad inhibition of these pathways may inadvertently impair immune-cell function (45).

Persistent antigen exposure can induce T-cell exhaustion, characterized by reduced effector function, sustained expression of inhibitory receptors, and transcriptional and epigenetic reprogramming (46, 47). Mitochondrial dysfunction and HIF-1α-related metabolic changes may drive the progression toward terminally exhausted T-cell states, consistent with evidence linking mitochondrial fitness to T-cell persistence (48, 49). An improved metabolic environment alone does not inherently reverse an exhaustion program, and PD-1/PD-L1 blockade may be insufficient to fully restore impaired T-cell function (47). Therefore, structural, cellular, and metabolic constraints inform evaluations of how AS-IV may alter local conditions relevant to checkpoint blockade, noting where proposed links remain indirect.

## 4 Potential modulation of physical barriers to tumor immune infiltration by AS-IV

Among the outlined barriers, AS-IV demonstrates endothelial-protective and antifibrotic activities pertinent to vascular and stromal constraints on immune-cell access. However, the robustness of evidence varies across these processes. Tumor-specific evidence currently supports modulation of CAF function (50, 51), while proposed effects on tumor vascular normalization, ECM remodeling, perfusion, and hypoxia largely depend on non-tumor or cross-context evidence (52–55) and multicomponent AS-IV-containing formulations. Such findings provide pharmacological rationale rather than direct evidence of tumor-specific TME normalization.

### 4.1 Vascular regulation of immune-cell trafficking

In a myocardial ischemia–reperfusion model, AS-IV reduced cardiac microvascular endothelial permeability (52), with related studies supporting endothelial-protective effects linked to the regulation of oxidative stress (53). While these findings justify investigating vascular barrier function in tumors, they do not prove that AS-IV normalizes tumor vasculature. Establishing this effect would require evidence of changes in vascular structure and maturity, perfusion, leakage, and oxygenation, along with assessments of whether these alterations are associated with modified CD8^+^ T-cell trafficking or spatial distribution within tumor tissue.

### 4.2 CAF- and ECM-related constraints on immune infiltration

More direct evidence from tumor contexts exists regarding CAF regulation. In one study, primary gastric CAFs (GCAFs) were isolated from a patient, revealing that AS-IV increased miR-214 and decreased miR-301a, alongside reductions in M-CSF production and secretion, and increases in TIMP2. Inhibition of miR-214 or overexpression of miR-301a, either alone or in combination, partially weakened the effects of AS-IV on the measured pro-tumor GCAF functions. Additionally, conditioned medium from AS-IV-treated GCAFs exhibited diminished capacity to promote BGC-823 cell proliferation, migration, and invasion (50). However, due to a known cell-line identity problem with BGC-823, caution is warranted in interpreting these findings specifically within a gastric cancer context. Collectively, these results suggest that AS-IV regulates GCAF secretory and pro-tumor functions through miR-214/M-CSF and miR-301a/TIMP2 pathways.

A separate co-culture study of CAFs implicated HOXA6/ZBTB12 regulation in AS-IV-induced suppression of CAF-supported tumor-cell growth, migration, and invasion (51). While these studies bolster the tumor-context evidence indicating that AS-IV can modify select CAF functions, they did not assess immune cells, ECM architecture or mechanics, or the spatial distribution of T cells *in vivo*. Consequently, they do not establish that AS-IV reduces stromal compression or reverses spatial immune exclusion.

Cross-context evidence supports a broader antifibrotic rationale. For example, in a renal fibrosis model, AS-IV diminished fibrotic remodeling and indicators related to fibronectin, vimentin, and α-SMA, aligning with existing evidence of antifibrotic activity beyond tumors (54, 55). Given the heterogeneity and context dependence of CAFs, general antifibrotic activity cannot be assumed to reflect selective modulation of CAF states associated with immune exclusion, and whether AS-IV alters tumor ECM organization, tissue mechanics, or CD8^+^ T-cell spatial distribution remains unresolved.

### 4.3 Hypoxia as a structural–immunometabolic bridge

Hypoxia serves as a mechanistic link between vascular and stromal abnormalities and immune dysfunction. Studies have shown that experimental reduction of tumor hypoxia correlates with improved outcomes in adoptive T-cell therapy. Additionally, metformin-mediated reduction of hypoxia has enhanced PD-1 blockade efficacy in mouse models (56, 57). These findings underscore the importance of examining whether changes in tumor perfusion or stromal architecture could impact immune function via alterations in oxygenation.

The vascular-protective and antifibrotic effects of AS-IV outside tumors present a testable hypothesis: improved vascular or stromal conditions may enhance perfusion, reduce hypoxia, and create a more favorable environment for immune-cell entry and function. While AS-IV-containing multicomponent platforms have reported changes in perfusion, hypoxia, and measures of tumor-infiltrating lymphocytes, attributing these effects to AS-IV alone remains problematic (58) (see Section 6.2). Thus, the proposed sequence linking vascular or stromal improvement to increased perfusion, reduced hypoxia, and enhanced immune-cell function remains a mechanistic hypothesis requiring integrated validation; changes in HIF-1α alone do not suffice.

Even if these physical constraints are alleviated, T cells that infiltrate the tumor may still face functional limitations imposed by suppressive immune-cell states and metabolic stress. These processes will be addressed in the following section.

## 5 Modulation of suppressive immune-cell states and immunometabolic constraints by AS-IV

Physical access represents only one requirement for effective antitumor immunity. Once T cells infiltrate tumor tissue, suppressive myeloid states, Treg-associated regulation, and metabolic stress can still impede their effector function (59–61). Studies involving AS-IV have investigated macrophage-associated states, Treg populations, CD8^+^ T-cell responses, and lactate-related metabolic constraints, though the strength of evidence varies across these areas. A key question, therefore, centers on whether AS-IV modifies specific cellular or metabolic conditions that affect T-cell function and responsiveness to checkpoint blockade.

### 5.1 Tumor-associated macrophages and myeloid regulation

Given that TAM function relies on cellular state as well as abundance, AS-IV-related modifications should be evaluated in terms of defined myeloid states and functions, rather than simplifying changes to a shift from “M2” to “M1” macrophages (32–35, 37). In lung cancer experiments, AS-IV reduced IL-4/IL-13-induced levels of CD206, Arg1, and associated suppressive factors in THP-1-derived macrophages, while conditioned medium from AS-IV-treated macrophages exhibited diminished capacity to promote migration and invasion of A549 and H1299 cells. AMPK-related interventions supported the involvement of this pathway (62, 63). However, M1-associated markers, such as TNF-α, iNOS, and COX-2, did not show significant increases *in vitro*, and the proportions of CD3^+^, CD4^+^, and CD8^+^ T cells remained unchanged *in vivo* (62, 63). These results indicate modulation of specific macrophage-associated states rather than a broad M2-to-M1 conversion.

In a separate CT26 study, AS-IV treatment of bone marrow-derived macrophages resulted in reduced measures related to CD206, Arg1, and Mrc1, while increasing levels of MHC-II, IL-12, and Nos2. Changes in intratumoral macrophage populations, characterized by CD206, MHC-II, and other markers, were also reported (14). Additional tumor studies support this evidence: in HCC co-culture experiments, AS-IV decreased M2-associated markers and macrophage-supported tumor-cell behaviors alongside alterations in TLR4/NF-κB/STAT3 signaling (64). Moreover, in colorectal cancer models, AS-IV reduced the release of tumor-derived extracellular vesicles and M2-associated macrophage measures, as well as liver metastasis (65). Notably, neither of these studies examined checkpoint blockade.

The combination studies involving CT26 and Lewis lung carcinoma further emphasize the significance of assay context. In the CT26 study, tumor macrophages were assessed using flow cytometry (14), while the Lewis study primarily utilized tissue CD86/CD206 immunofluorescence without macrophage-lineage co-staining (15). Although both support myeloid-associated changes within the tested conditions, neither establishes that these changes are causally required for observed combination benefits.

Other myeloid functions remain less clearly defined. PD-1 expression on TAMs has been linked to impaired phagocytosis in some models, and checkpoint blockade can enhance macrophage phagocytic activity (66); however, this mechanism has yet to be tested in AS-IV studies. Direct evidence regarding the effects of AS-IV on intratumoral MDSCs is also limited. Consequently, more definitive studies will necessitate functional and causal approaches that differentiate changes in myeloid-cell markers from those affecting phagocytosis, T-cell suppression, or contributions to ICI responsiveness.

### 5.2 Treg-related suppression and T-cell responses

Evidence indicating that AS-IV directly modifies Treg-mediated suppression in tumors remains limited. Changes in the Th17/Treg balance observed in experimental colitis provide cross-context evidence of immune modulation (67). In an orthotopic lung cancer model employing IDO-overexpressing 3LL cells, oral AS-IV reduced the proportion of splenic CD4^+^CD25^+^ cells while increasing the proportion of splenic CD8^+^CD28^+^ cells, along with decreased IDO expression (68). These findings pertain to peripheral T-cell populations and do not confirm reduced suppressive function of intratumoral Tregs; additionally, an increased CD8^+^CD28^+^ proportion does not, by itself, demonstrate enhanced cytotoxic function.

Tumor-cell co-culture studies offer more direct connections between AS-IV-associated signaling and CD8^+^ T-cell responses. In nasopharyngeal carcinoma, AS-IV was linked to reduced SATB2/Wnt/β-catenin/PD-L1-related signaling. In tumor-cell–CD8^+^ T-cell co-cultures, increased LDH release and IFN-γ levels were observed, along with elevated perforin and granzyme B levels in the recovered CD8^+^ T cells; interventions targeting the SATB2 and Wnt pathways further supported this signaling relationship (17). In prostate cancer, perturbation of PPP1R14B, along with chemotaxis and co-culture experiments, connected Wnt/β-catenin/CX3CL1 signaling to CD8^+^ T-cell responses (13). Notably, AS-IV applied directly to isolated CD8^+^ T cells did not significantly elevate IFN-γ under the tested conditions, whereas the corresponding response was evident in tumor-cell co-culture. This observation aligns with the broader hypothesis that part of the immune effect of AS-IV may derive from modifications to tumor-cell or local microenvironmental conditions rather than from nonspecific stimulation of T cells.

Nonetheless, these T-cell-related changes must be distinguished from the reversal of established exhaustion. Exhaustion entails persistent functional impairment alongside transcriptional and epigenetic reprogramming, and cannot be concluded solely from a decrease in PD-1 expression, an increase in T-cell abundance, or a single effector-associated readout (46, 47, 69–71). Current AS-IV studies provide evidence of changes in T-cell subset proportions, chemotaxis, and cytotoxicity- or effector-related measures under specific experimental conditions. An inhaled dual-drug formulation containing AS-IV has also been associated with changes in exhaustion-related markers and effector-associated populations (72) (see Section 6.2); however, these findings do not confirm the reversal of a pre-existing exhaustion program. Whether the tumor-cell-mediated T-cell responses described above contribute directly to additional benefits from checkpoint blockade remains to be determined.

### 5.3 Immunometabolic modulation

Among the local conditions influencing T-cell function, metabolic stress stands out as one of the more mechanistically developed areas of AS-IV-related evidence. Cross-context studies indicate that AS-IV can affect metabolic and inflammatory programs: in AOM/DSS-induced colitis-associated tumorigenesis, SPR supported an interaction between AS-IV and PPARγ, while GW9662 intervention implicated related signaling pathways (73). A non-tumor ischemic injury study proposed a mechanism involving HIF-1α/GABA-T/succinate (74). These findings lend support to the metabolic plausibility of AS-IV but do not demonstrate relief from nutrient competition or restoration of T-cell metabolic fitness in established tumors.

The pertinent pharmacological question is not whether AS-IV broadly alters tumor metabolism, but whether it alleviates specific metabolic constraints that suppress immune-cell function. A more direct example arises from studies in oral squamous cell carcinoma, where AS-IV treatment correlated with reduced MCT1 expression, lower lactate levels, decreased extracellular acidification, and diminished tumor-cell surface PD-L1 in tumor-cell–CD8^+^ T-cell co-cultures. Concurrently, LDH release and levels of IFN-γ, TNF-α, granzyme B, and perforin increased (75). Exogenous lactate was found to weaken the CD8^+^ T-cell-related antitumor effects, while AS-IV partially countered these lactate-associated changes; MCT1 overexpression further indicated involvement of this axis. In tumor-bearing mice, tumor growth, tissue lactate, and MCT1-related measures decreased, while CD8^+^ staining in tumor tissue increased. This study thereby connects an AS-IV-associated alteration in the tumor lactate environment with functional measures related to T cells within the same experimental framework, although checkpoint blockade was not examined (75).

These findings should be interpreted in light of the broader complexity of tumor lactate metabolism. Lactate production via LDHA and lactate transport through MCTs represent distinct intervention points (41, 42), so a reduction in lactate following AS-IV treatment does not confirm inhibition of LDHA. Similarly, LDH release in a cytotoxicity assay does not serve as a proxy for LDHA enzymatic activity. Lactate and lactylation can exert context-dependent effects on T cells, and both tumor and immune cells share several metabolic requirements (43, 45).

In summary, the current findings primarily suggest modifications to the extracellular lactate environment rather than demonstrating altered protein lactylation, direct metabolic reprogramming of T cells, or reversal of an existing exhaustion program. Future studies should clarify tumor-cell-mediated alterations in the extracellular metabolic environment versus direct effects of AS-IV on immune-cell metabolism, and determine whether either mechanism contributes to enhanced benefits with checkpoint blockade.

## 6 Molecular mechanisms and direct evidence relevant to ICI responses

Beyond physical and cellular constraints, chemokine, metabolic, and checkpoint signaling further influence T-cell recruitment and function within tumors. The available evidence can thus be assessed based on mechanistic depth and proximity to checkpoint-blockade outcomes (**Figure 2**).

**Figure 2:**
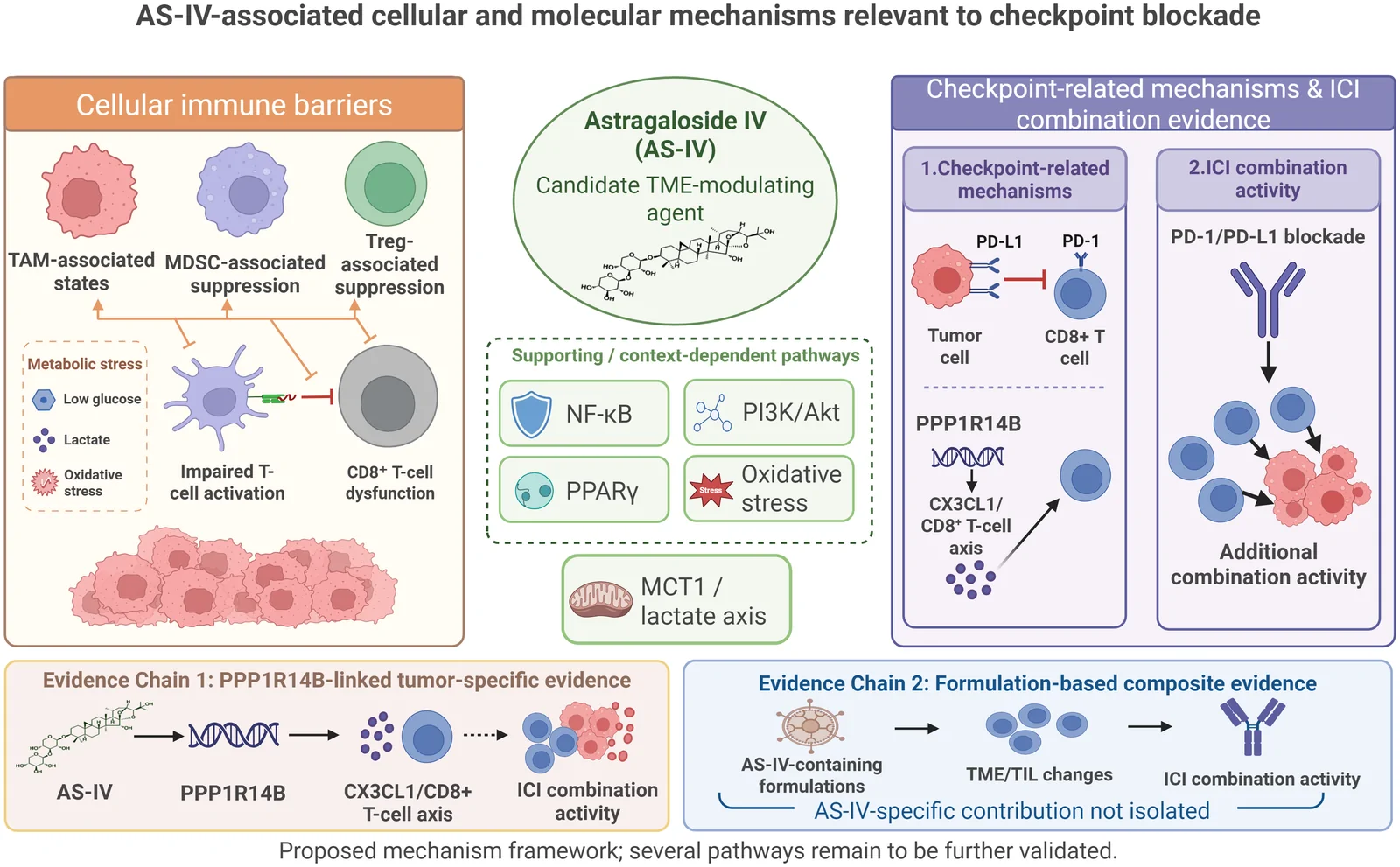
Cellular and molecular evidence linking AS-IV to mechanisms relevant to checkpoint blockade. AS-IV has been associated with changes in macrophage-, Treg-, myeloid-, and T-cell-related states, together with context-dependent signaling and metabolic effects. The PPP1R14B/CX3CL1/CD8+ T-cell axis provides the most coherent evidence chain linking AS-IV target engagement, downstream immune-associated signaling, and anti-PD-1 treatment outcomes, although its requirement for the additional combination benefit has not been established. The MCT1/lactate axis provides tumor-specific target-interaction and functional evidence, but checkpoint blockade was not tested. PD-L1 regulation is context dependent and should not be interpreted as a uniform directional effect of AS-IV. NF-κB, PI3K/Akt, PPARγ, and oxidative-stress-related pathways are shown as supporting or context-dependent mechanisms. AS-IV-containing formulations provide additional proof-of-concept for checkpoint-based combination strategies, but their composite design prevents attribution of the observed effects to AS-IV alone. Dotted arrows indicate mechanistic links that remain incompletely validated. Created in https://BioRender.com.

### 6.1 Mechanistic evidence: the PPP1R14B/CX3CL1/CD8^+^ T-cell and MCT1/lactate axes

The efficacy of checkpoint blockade relies not only on reversing inhibitory signaling but also on the presence and functional competence of effector T cells within the tumor. In this context, the prostate cancer study linking AS-IV-associated tumor-cell signaling to CX3CL1 and CD8^+^ T-cell responses represents one of the more mechanistically resolved examples in the current evidence base (13).

SPR experiments with recombinant PPP1R14B supported binding of AS-IV, with a dissociation constant of 4.88 μM. CETSA findings in PC-3 and DU-145 cells supported target engagement in a cellular environment, while molecular docking suggested a potential binding conformation (13). Protein stability and ubiquitination experiments, along with reversal by the proteasome inhibitor MG-132, implicated the ubiquitin–proteasome pathway in the degradation of PPP1R14B. Overexpression, knockdown, and downstream interventions further linked PPP1R14B to Wnt/β-catenin, CX3CL1, and CD8^+^ T-cell-related responses. Additionally, AS-IV improved tumor control during anti-PD-1 treatment in an RM1 model, thereby connecting compound–target interaction, downstream immune-associated signaling, and checkpoint-blockade treatment outcomes within the same research framework.

Nevertheless, important causal steps remain unresolved. Binding-site perturbation has not clarified whether AS-IV–PPP1R14B binding is necessary to initiate PPP1R14B degradation, and the responsible E3 ubiquitin ligase has yet to be identified. AS-IV monotherapy and ICI combination treatment were examined in separate experiments rather than in a matched four-arm design. The necessity of this axis for additional combination benefits has not been established through CD8^+^ T-cell depletion or CX3CL1/CX3CR1 blockade. Also unresolved are the relevant cellular sources and responding populations *in vivo*, along with any redistribution of CD8^+^ T cells from tumor margins or stroma into tumor parenchyma. Findings linking PPP1R14B to tumor progression or treatment response in other contexts (76) do not confirm conservation of this immune mechanism across tumor models.

A second target-interaction mechanism has been identified in oral squamous cell carcinoma. MST and SPR detected interactions between AS-IV and MCT1, yielding dissociation constants of approximately 0.89 and 2.57 μM, respectively. Photoaffinity pull-down assays in cell lysates provided additional evidence of interaction (75). MCT1 overexpression, along with changes in lactate, extracellular acidification, PD-L1, and CD8^+^ T-cell-related functional readouts, further implicated this axis. The study also noted reduced MCT1 mRNA stability, although the relationship between this observation and the AS-IV–MCT1 interaction remains unclear. Changes in lactate levels do not confirm direct inhibition of MCT1 transport. Evidence of target engagement in intact cells and proof that MCT1 is essential for *in vivo* efficacy would further bolster causality. Importantly, checkpoint blockade was not tested; thus, the MCT1/lactate axis offers compelling tumor-specific mechanistic evidence relevant to ICI responsiveness but does not constitute direct evidence of enhanced benefits from combining AS-IV with checkpoint blockade.

### 6.2 Direct combination studies and evidence from formulations

Beyond the prostate model, five additional studies directly tested free AS-IV in conjunction with PD-1/PD-L1 blockade (14–18). In the CT26 colorectal cancer model, AS-IV combined with anti-PD-1 exhibited improved tumor control compared to either monotherapy, along with a higher proportion of CD8^+^ cells among CD45^+^ tumor-derived cells and changes in the cytokine environment (14). In one Lewis lung carcinoma study, the combination treatment also enhanced tumor control; however, immune assessment primarily relied on tissue immunofluorescence for CD3, CD69, CD86, and CD206 instead of functional assays for CD8^+^ T cells (15). In a second Lewis study, Meng et al. similarly reported greater tumor growth inhibition with the combination and higher proportions of CD3^+^CD8^+^ cells in tumor-derived lymphocyte preparations, although the parent population and full gating strategy were not specified (18). Collectively, these findings support additional combination activity but do not confirm restoration of T-cell effector function or reversal of an established exhaustion program.

In the 4T1 triple-negative breast cancer model, AS-IV combined with anti-PD-L1 improved tumor control and was associated with increases in CD3^+^CD8^+^, CD8^+^IFN-γ^+^, and CD8^+^granzyme B^+^ populations, although the parent populations and full gating strategy were inadequately specified (16). Tumor-cell experiments further implicated STING/TBK1-related signaling: AS-IV increased p-STING, p-TBK1, and PD-L1-associated measures, and responses were attenuated by H-151. These data support the involvement of STING-associated signaling but do not demonstrate direct binding of AS-IV to cGAS or STING, nor establish that this pathway is required for the *in vivo* combination benefit. Additionally, the relatively high AS-IV concentration used in principal *in vitro* experiments significantly reduced cell viability, necessitating interpretation of the signaling findings alongside cytotoxicity. A study in nasopharyngeal carcinoma also reported AS-IV–checkpoint blockade activity; however, the identity, product information, and dosage of the *in vivo* checkpoint antibody were inconsistently or incompletely reported (17) (see **Table 1**).

AS-IV-containing formulations constitute a distinct level of evidence, as their effects cannot be attributed solely to free AS-IV. A homologous membrane-coated magnetic MOF co-loaded with tanshinone IIA and AS-IV in HCC, an oxymatrine/AS-IV liposomal system in 4T1-Luc tumors, and a platelet membrane-coated magnetic MOF co-delivering oxymatrine and AS-IV in HCC demonstrated additional activity when combined with anti-PD-1, alongside changes in vascular, stromal, TIL, or metabolic measures (58, 77, 78). The liposomal study also quantified plasma and tumor-tissue AS-IV exposure, providing valuable pharmacokinetic data. However, these multicomponent systems lacked sufficiently matched single-component controls to isolate the contribution of AS-IV.

Two further studies explored alternative delivery strategies. An inhaled dual-drug liposome containing AS-IV and polyphyllin VII improved anti-PD-1-associated tumor control and modified T-cell-related measures in a lung tumor model (72). In contrast, AS-IV-loaded micelles with surface-conjugated anti-PD-L1 showed improved tumor control compared to AS-IV-loaded micelles followed by free anti-PD-L1 in an orthotopic bladder cancer model (79). These studies support the feasibility of integrating AS-IV into checkpoint-based delivery strategies; however, variations in drug composition, carrier design, antibody presentation, or administration sequence hinder the isolation of an AS-IV-specific effect.

Overall, studies involving free AS-IV demonstrate greater combination activity across several preclinical tumor models, while formulation studies support the feasibility of AS-IV-containing checkpoint-based delivery strategies. None of these studies reported formal pharmacological synergy analysis using a defined reference model. Thus greater activity than corresponding monotherapies does not establish pharmacological synergy. Additionally, pretreatment immune-desert or spatially immune-excluded phenotypes were not adequately characterized in most models, failing to demonstrate phenotype-specific benefits or “cold-to-hot” conversion. Similarly, the inhaled formulation study did not establish acquired anti-PD-1 resistance before treatment, preventing demonstration of reversal of acquired resistance. These limitations emphasize the importance of interpreting checkpoint-related molecular changes, particularly regarding PD-L1, within their specific experimental contexts.

### 6.3 Context-dependent regulation of PD-L1

The available tumor-specific evidence does not support a uniform directional effect of AS-IV on PD-L1. In HCC cells, a miR-135b-5p/CNDP1-related mechanism was linked to reduced IFN-γ-induced cell-surface PD-L1 and increased tumor-cell killing in PBMC co-culture (80). In nasopharyngeal carcinoma, reductions in PD-L1 mRNA and total protein were associated with the suppression of SATB2/Wnt/β-catenin signaling (17). In oral squamous cell carcinoma–CD8^+^ T-cell co-culture, MCT1/lactate-related changes and lower acidification corresponded with decreased tumor-cell surface PD-L1 and enhanced effector-related readouts (75).

Conversely, AS-IV treatment in triple-negative breast cancer cells increased STING/TBK1-related signaling, IFN-β transcription, and total PD-L1 protein, while AS-IV combined with anti-PD-L1 improved tumor control *in vivo* (16). One possible interpretation is that increased PD-L1 reflects inhibitory feedback associated with a more inflammatory STING-mediated state, although upregulation of PD-L1 has not been shown to be necessary for the additional combination benefit.

These divergent findings indicate that PD-L1 should be interpreted in context rather than as a unidirectional pharmacodynamic marker of AS-IV action. Variations in stimulation conditions, AS-IV exposure, upstream signaling, cellular sources, and assay types exist across studies; furthermore, cell-surface PD-L1, total protein, and mRNA are distinct measurements. Therefore, the evidence supports context-dependent regulation of PD-L1 rather than uniform suppression or induction. Changes in PD-L1 alone are insufficient to define the degree of immune suppression or predict the benefit of AS-IV in combination with ICIs.

### 6.4 Evidence hierarchy and boundaries

Overall, the evidence can be assessed based on its proximity to the proposed AS-IV–ICI relationship. Direct combinations of free AS-IV with PD-1/PD-L1 blockade provide the most direct evidence of combination activity, although the mechanistic depth varies significantly among studies. Tumor-specific studies lacking direct ICI testing offer complementary mechanistic insights, with the PPP1R14B/CX3CL1/CD8^+^ T-cell and MCT1/lactate axes being the most clearly resolved examples. AS-IV-containing formulations lend additional proof-of-concept for checkpoint-based combination strategies, but they cannot isolate the contribution of AS-IV from that of co-delivered compounds or delivery systems. Proposed effects on tumor vascular normalization, ECM decompression, hypoxia relief, and other TME processes are primarily supported by cross-context pharmacology, warranting their consideration as mechanistic hypotheses requiring direct tumor-specific validation.

Across these evidence levels, treatment activity, immune phenotype changes, and compound–target interaction represent distinct levels of inference: greater antitumor activity does not automatically establish pharmacological synergy, post-treatment immune changes do not confirm the conversion of a specific pretreatment phenotype, and compound–target interaction alone does not confirm causal necessity for therapeutic benefit. AS-IV should therefore be viewed as a candidate TME-modulating adjunct rather than as an established component of ICI combination therapy. Remaining critical questions pertain to causal validation, pharmacologically relevant exposure, treatment timing and delivery, responsive TME phenotypes, and safety; these translational issues are explored in Section 7.

## 7 Translational challenges and validation strategies for combining AS-IV with ICIs

Although AS-IV exhibits pharmacological activities relevant to several determinants of ICI responsiveness, current oncological evidence remains preclinical and heterogeneous in mechanistic depth. The central pharmacological and translational questions include whether the proposed effects are causal in tumors, achievable at pharmacologically relevant exposures, reproducible in defined TME contexts, and compatible with an acceptable efficacy–safety window.

### 7.1 Evidence quality and causal validation

Direct preclinical studies indicate enhanced activity when AS-IV is combined with PD-1/PD-L1 blockade; however, methodological quality and reporting vary across studies. Some experiments included appropriate monotherapy and combination controls, and several incorporated target-interaction, genetic, or functional validation. Nonetheless, aspects such as randomization, blinding, sample-size justification, and units of independent replication were not consistently reported. These limitations diminish confidence in the robustness of individual findings, as summarized in **Table 1** and **Supplementary Table 2**.

To establish mechanistic causality, testing should involve target or pathway perturbation and, where applicable, immune-cell depletion or reconstitution. A stronger evidence chain would connect AS-IV exposure to a specific target or cell state, subsequent TME changes, corresponding immune-cell function, and the additional benefit from PD-1/PD-L1 blockade. Demonstrating a loss of combination benefit following disruption of the proposed mechanism would provide significantly stronger evidence of causality than phenotypic associations alone.

### 7.2 Pharmacokinetics, treatment timing, and delivery

Pharmacokinetic feasibility poses another major challenge. AS-IV tissue distribution has primarily been characterized in animal models (81), while the available human study examined intravenous total Astragalus saponins in healthy volunteers rather than purified AS-IV or an AS-IV–ICI regimen. Across tested infusion regimens, mean peak plasma AS-IV concentrations ranged from 2.12 to 5.17 μg/mL (approximately 2.70 to 6.59 μM), with an elimination half-life of 2.14 to 2.69 hours (82). These data provide a quantitative reference for human exposure to that preparation but do not confirm effective exposure in tumors or target cells.

Nominal AS-IV concentrations in several key cell experiments exceeded this human plasma reference. In the prostate cancer study, 40 μM corresponds to approximately 31.4 μg/mL, which is about 6.1 times the mean peak plasma concentration in the highest-exposure group of the human study (13, 82). In the TNBC and HCC studies, the 80 μg/mL used corresponds to approximately 101.9 μM, or about 15.5 times that reference concentration (16, 80, 82). These comparisons indicate differences in concentration scale; however, the experimental concentrations are not necessarily minimum effective concentrations, and the reported human Cmax does not define an upper achievable exposure limit.

The PPP1R14B and MCT1 studies reported target-interaction evidence but did not measure plasma or tumor AS-IV exposure under their respective *in vivo* dosing regimens (13, 75). Binding constants alone do not confirm whether relevant target exposure was attained *in vivo*. Additional uncertainty surrounds the active molecular species: orally administered AS-IV may undergo gut microbiota-mediated deglycosylation to metabolites such as cycloastragenol (83). Future studies should therefore quantify both parent AS-IV and major metabolites in plasma and tumor tissue under the same dosing regimens used for efficacy experiments, and relate exposure to target and immune pharmacodynamic readouts.

Treatment timing may significantly affect efficacy. If AS-IV modifies vascular, stromal, or immune conditions, these effects may need to precede or coincide with checkpoint blockade. Dose- and time-dependent effects observed with other vascular-modulating interventions provide a rationale for considering treatment sequence (84, 85). However, comparable windows for AS-IV have not been established, and pretreatment has not been demonstrated to be superior to concurrent treatment. Therefore, dose, sequence, and schedule require direct comparison.

Delivery methods also play a critical role. Limitations in systemic exposure and heterogeneous tumor perfusion may restrict intratumoral availability of AS-IV. Nanodelivery can enhance stability and distribution, yet it does not guarantee effective tumor penetration (86, 87). The HCC TSA/AS-IV@MOF study supports a delivery-based combination concept (58), but the contribution of AS-IV cannot be isolated from that of the co-administered drug and carrier. A second formulation study indicated that oxymatrine/AS-IV co-loaded liposomes increased AS-IV exposure and improved anti-PD-1-associated tumor control in a breast cancer model (77). Relative to the free-drug combination, plasma AUC_0_–_24_ h and Cmax were approximately 2.04-fold and 1.22-fold higher, respectively, while tumor AUC_0_–_24_ h and Cmax were approximately 2.53-fold and 4.17-fold higher. However, because oxymatrine and AS-IV were co-delivered within the same formulation, the therapeutic contribution of increased AS-IV exposure cannot be disentangled from that of the co-administered compound and delivery system.

The inhaled dual-drug liposome study also provided tissue-distribution data. At 24 hours post-dosing, HPLC measurements showed higher AS-IV concentrations in lung tissue with the tested inhaled regimen compared to oral administration (72). While this single measurement supports a difference in local exposure under those conditions, it does not replace plasma or tumor concentration–time profiles nor establish exposure within the relevant target cells.

### 7.3 Candidate biomarkers and future patient stratification

The effects of AS-IV are unlikely to be uniform across different tumors. If AS-IV-based combinations are to be developed further, candidate TME features for prospective evaluation may include vascular and stromal states, myeloid-cell states, CD8^+^ T-cell density and spatial distribution, and characteristics related to PPP1R14B/CX3CL1 or MCT1/lactate (13, 75). While PD-L1 may provide complementary information, it is unlikely to serve as a sufficient standalone marker.

Prospective validation should differentiate poorly infiltrated and spatially immune-excluded phenotypes from T-cell dysfunctional states, as the relevant barriers and candidate biomarkers are likely not identical across contexts. Pretreatment phenotypes should be defined independently of post-treatment immune changes or treatment responses.

Currently, none of these features has been established as predictive biomarkers for the benefit of adding AS-IV. Future studies should measure candidate markers prior to treatment and directly compare ICI monotherapy with the combination to ascertain whether they predict additional benefit; post-treatment changes should be interpreted primarily as pharmacodynamic readouts. Single-cell and spatial approaches may facilitate discovery, but functional validation in independent models or cohorts remains essential.

### 7.4 Context dependence and the balance between efficacy and safety

The pleiotropic actions of AS-IV may be context-dependent. Anti-inflammatory activity could be beneficial when it reduces tumor-promoting inflammation but may also suppress antitumor immune activation under certain conditions. Similarly, broad modulation of fibroblasts or macrophages may impact both tumor-promoting and tumor-restraining populations. Thus, factors such as dose, treatment timing, tumor context, and pretreatment TME phenotype may influence both the direction and magnitude of benefit.

Available safety findings primarily concern tolerability observed under specific animal dosing regimens and formulations. Two studies of multicomponent platforms containing AS-IV reported no significant major organ pathology or abnormalities in routine liver and kidney biochemistry during their observation periods (58, 77). Greater *in vitro* hemolysis was noted with corresponding free-drug combinations than with loaded formulations, indicating that formulation can influence the observed safety profile. These findings pertain to the complete interventions tested and cannot isolate the safety contribution of AS-IV itself or establish the long-term safety of AS-IV–ICI combinations.

The inhaled AS-IV/polyphyllin VII liposome study provided additional route-specific safety information. Acute hematological and biochemical measures were assessed in a separate mouse experiment, with no evident respiratory-tract abnormalities reported following repeated inhalation under the tested regimen (72). However, anti-PD-1 was not included in that safety experiment, meaning these findings primarily inform the tolerability of the tested formulation and inhalation regimen rather than the safety of the ICI combination.

An intravenous AS-IV study involving pregnant rats and rabbits reported increased fetal deaths, suggesting that general tolerability findings cannot substitute for developmental safety assessments (88). A study involving healthy volunteers who received intravenous total Astragalus saponins provided short-term human pharmacokinetic and tolerability data, reporting mild, self-resolving adverse events, including increases in bilirubin and rash (82). However, these findings cannot be directly extrapolated to AS-IV administered to patients with cancer or in combination with checkpoint blockade. No published human oncology study evaluating AS-IV as an intervention was identified through literature searches. A targeted search of ClinicalTrials.gov, the WHO ICTRP, and the ChiCTR revealed no registered human oncology trial evaluating AS-IV itself as an intervention, either alone or in combination with another anticancer treatment, including PD-1/PD-L1 blockade, as of 7 October 2026. Consequently, current evidence does not establish the clinical safety or antitumor efficacy of AS-IV–ICI combinations.

Safety and efficacy should be evaluated independently. ICI-related adverse events can affect multiple organs and may be persistent or severe (89, 90), while ICI-associated liver injury manifests heterogeneously (91, 92). The anti-inflammatory or organ-protective effects of AS-IV should not be interpreted as evidence that it prevents or treats these adverse events. Future combination studies should therefore assess antitumor activity and safety in parallel, utilizing the intended route, formulation, and repeated-dosing schedule, with pharmacokinetic/pharmacodynamic (PK/PD) analyses to relate exposure to efficacy and toxicity. Additionally, pharmaceutical quality, formulation-specific risks, and potential interactions with concomitant treatments will need thorough evaluation before clinical development.

Host defense against infection also merits consideration. In murine *E. coli* peritonitis, AS-IV promoted neutrophil recruitment and bacterial clearance (93). In murine polymicrobial sepsis, it reduced bacterial burden, circulating inflammatory mediators, and lymphocyte apoptosis (94). In a separate sepsis study, AS-IV reduced neutrophil extracellular trap (NET) formation and inflammatory injury, with mechanistic evidence implicating IκBα/NF-κB signaling, although pathogen clearance was not assessed (95). In coxsackievirus B3-infected mice, AS-IV reduced viral titers, accompanied by increased IFN-γ mRNA expression (96). Collectively, these studies do not support a uniformly immunosuppressive effect of AS-IV across infection models. However, they do not establish whether AS-IV can limit inflammatory injury during cancer therapy or checkpoint blockade without compromising either pathogen control or antitumor immune function.

## 8 Discussion and outlook

At present, AS-IV is best regarded as a candidate modulator of the tumor immune microenvironment, with its role still insufficiently validated. The evidence base has expanded beyond cross-context pharmacological plausibility: tumor-specific studies indicate that AS-IV is associated with the modulation of macrophage and CAF function, lactate-related metabolic suppression, checkpoint-associated signaling, and CD8^+^ T-cell responses. Direct combinations of free AS-IV with PD-1/PD-L1 blockade have been reported across various tumor models (13–18). However, these observations vary widely in evidential strength. The PPP1R14B/CX3CL1/CD8^+^ T-cell axis currently offers the most coherent chain linking compound–target interaction, immune-associated signaling, and checkpoint-blockade treatment outcomes. Meanwhile, the MCT1/lactate axis provides robust tumor-specific mechanistic evidence, albeit without direct testing in checkpoint blockade. Formulation-based studies offer additional proof-of-concept; however, they involve composite interventions, making it difficult to attribute effects solely to AS-IV (58, 72, 77–79). This hierarchy supports the consideration of AS-IV as a potential partner in ICI therapies, while retaining the distinction among direct combination evidence, tumor-specific mechanistic explorations, and formulation-based findings.

Conceptually, the potential value of AS-IV may be less about broadly stimulating antitumor immunity and more about modifying the local conditions under which checkpoint blockade operates. In tumors that are poorly infiltrated or spatially immune-excluded, checkpoint inhibition may be limited due to insufficient effector T cell access to critical tumor compartments, while in T cell-infiltrated tumors, local suppressive or metabolic constraints may impede effector function. Within this framework, AS-IV may be better characterized as a context-modifying agent that may modify local conditions influencing immune-cell access or functional competence, rather than a conventional systemic immune stimulant. However, since the pretreatment spatial immune phenotype was inadequately characterized in most combination models, this framework should not be interpreted as evidence of a general “cold-to-hot” conversion.

The divergent effects on PD-L1 regulation—with reductions observed in HCC, NPC, and OSCC but STING-mediated upregulation in TNBC—argue against a singular, conserved checkpoint mechanism for AS-IV (16, 17, 75, 80). Furthermore, the broad pharmacological profile of AS-IV should not imply that vascular, stromal, myeloid, metabolic, and checkpoint-related mechanisms contribute equally to combination benefits. Their relevance likely depends on tumor context, the cellular compartment in which AS-IV acts, and whether any observed change is causally linked to treatment response.

Pharmacological causality presents another layer of uncertainty. The biologically relevant *in vivo* species may not be restricted to parent AS-IV, especially after oral administration, where gut microbiota-mediated metabolism can produce downstream metabolites such as cycloastragenol (83). Concurrently, tumor-specific exposure to both parent AS-IV and its relevant metabolites remains somewhat undefined, and formulation-dependent delivery may further modify local drug distribution. Thus, mechanisms observed with parent AS-IV *in vitro* cannot automatically be assumed to reflect the active molecular species or exposure profile driving *in vivo* tumor responses.

Future studies should prioritize tumor-specific causal validation, the identification of immune-cell populations necessary for additional combination benefits, the definition of pharmacologically relevant tumor exposure, and the selection of responsive TME contexts. These considerations should be explored alongside dosing, treatment schedule, and safety to establish an acceptable therapeutic window. AS-IV should therefore be regarded not as an established component of ICI combination therapy, but as a testable candidate for TME modulation, with its translational value hinging on rigorous evidence linking drug exposure, tumor-specific mechanisms, immune context, and treatment efficacy.
