# Supplementary material for: Astragaloside IV and tumor microenvironmental barriers: immune mechanisms, evidence hierarchy, and implications for checkpoint blockade
Source: Front Immunol. 2026 Oct 22;17:1991297. doi: 10.3389/fimmu.2026.1991297 (PMC13735963; doi:10.3389/fimmu.2026.1991297)
Supplement: Supplementary file 1 [file Table1.docx]

**Supplementary Table S1. Literature-search strategy, trial-registry search, evidence-selection framework, and operational definitions**

**S1A. Bibliographic database search strategy**

| **Database** | **Coverage** | **Search date** | **Search field** | **Exact search string** | **Records retrieved** |
| --- | --- | --- | --- | --- | --- |
| PubMed/  MEDLINE | Inception to 6 Oct 2026 | 6 Oct 2026 | Title/Abstract | ("Astragaloside IV"[Title/Abstract] OR "Astragaloside-IV"[Title/Abstract]) AND (cancer*[Title/Abstract] OR tumor*[Title/Abstract] OR tumour*[Title/Abstract] OR neoplasm*[Title/Abstract] OR carcinoma*[Title/Abstract] OR malignan*[Title/Abstract] OR tumorigenesis[Title/Abstract] OR tumourigenesis[Title/Abstract]) | 301 |
| Web of Science Core Collection | Inception to 6 Oct 2026 | 6 Oct 2026 | Topic | ("Astragaloside IV" OR "Astragaloside-IV") AND (cancer* OR tumor* OR tumour* OR neoplasm* OR carcinoma* OR malignan* OR tumorigenesis OR tumourigenesis) | 168 |

The two database searches yielded 469 records. Records were imported into Zotero, and cross-database duplicates were identified using Zotero’s duplicate-detection function and manually verified where necessary. A total of 128 duplicate records were removed, leaving 341 unique records. Reference lists of relevant primary studies and recent reviews were additionally screened for eligible publications. Supporting evidence concerning non-tumor AS-IV pharmacology, pharmacokinetics, safety, and cross-context mechanistic plausibility was identified separately through targeted searches and citation tracking. Records identified through these supporting searches were used for contextual interpretation and were not included in the 469-record bibliographic-search count or the 20-study principal evidence framework unless they independently met the criteria for Categories A–C. Because this was a critical narrative Review and Category B was defined as a representative rather than an exhaustive mechanistic evidence stratum, the 341 unique records should not be interpreted as a formal PRISMA-style screening denominator from which the 20 principal studies were selected through an exhaustive inclusion process.

**S1B. Clinical-trial registry search**

For the registry search, an eligible study was defined as a human oncology study in which AS-IV itself was administered as an intervention, either alone or in combination with another anticancer treatment, including immune checkpoint blockade. Studies of Astragalus-containing herbal preparations, multicomponent products in which AS-IV was not the defined intervention, studies in which AS-IV was measured only as a pharmacokinetic constituent, and non-oncology studies were not considered eligible AS-IV oncology trials.

| **Registry** | **Search date** | **Search terms used** | **Search restrictions** | **Search outcome** | **Eligible human oncology trials evaluating AS-IV** |
| --- | --- | --- | --- | --- | --- |
| ClinicalTrials.gov | 7 October 2026 | Astragaloside IV; Astragaloside-IV; Astragaloside 4; AS-IV; Astragaloside | All study statuses; no date restriction | AS-IV/astragaloside- and Astragalus-related records were identified, including non-oncology studies and oncology studies involving Astragalus-based preparations, but no oncology trial evaluating AS-IV itself as the intervention was identified | 0 |
| WHO International Clinical Trials Registry Platform (ICTRP) | 7 October 2026 | Astragaloside IV; Astragaloside-IV; Astragaloside; AS-IV | No recruitment-status restriction | No eligible AS-IV oncology record was identified | 0 |
| Chinese Clinical Trial Registry (ChiCTR) | 7 October 2026 | 黄芪甲苷IV; 黄芪甲苷 IV; 黄芪甲苷Ⅳ; 黄芪甲苷; Astragaloside IV; Astragaloside-IV | No recruitment-status restriction | AS-IV-related records were identified, but the relevant records concerned non-oncology indications | 0 |

The ChiCTR search identified AS-IV-related records including a study of endometriosis-associated fibrosis and a phase III study of astragaloside glucose injection for stable exertional angina; both were excluded because they were non-oncology studies. No published human oncology study evaluating AS-IV itself as an intervention was identified in the bibliographic literature search, and no eligible registered human oncology trial was identified in the three trial registries searched.

**S1C. Evidence-selection and study-counting framework**

| **Evidence category** | **Operational inclusion logic** | **Counting rule** | **Number of principal studies** |
| --- | --- | --- | --- |
| Category A. Direct combinations of free AS-IV with ICIs | Primary tumor studies directly testing free AS-IV together with PD-1/PD-L1 blockade | Eligible direct combination studies were counted individually | 6 |
| Category B. Representative tumor-specific mechanistic evidence | Primary tumor-specific studies of free AS-IV addressing distinct checkpoint-relevant immune, stromal, or metabolic mechanisms without direct ICI testing | Representative, non-redundant mechanistic stratum; not intended as an exhaustive catalog of all tumor-related AS-IV studies | 9 |
| Category C. ICI combination studies using AS-IV-containing formulations or delivery systems | Studies testing AS-IV-containing multicomponent formulations or delivery systems together with checkpoint blockade | Eligible formulation/delivery studies were counted individually and were not interpreted as equivalent to free-AS-IV combination evidence | 5 |
| Cross-context pharmacological evidence | Non-tumor or broader pharmacological studies used to support mechanistic plausibility, pharmacokinetics, safety, or translational interpretation | Supporting evidence only; not counted among the 20 principal studies | Not applicable |

Categories A–C were mutually exclusive for study counting. Correction notices were linked to the corresponding primary study rather than counted as independent studies. For Category B, priority was given to studies representing distinct checkpoint-relevant mechanisms involving T-cell responses, checkpoint regulation, stromal or macrophage-associated states, and immunometabolic constraints. Closely overlapping mechanistic studies were treated as corroborative evidence rather than counted separately. Category B was therefore intended to provide mechanistic breadth without implying exhaustive inclusion of all tumor-related AS-IV studies.

**S1D. Operational definitions used for evidence interpretation**

| **Term** | **Operational definition** |
| --- | --- |
| **Pretreatment immune phenotype** | Classified only from baseline or pretreatment measurements reported for the specific model. Tumor type, treatment response, or post-treatment immune changes were not used to infer the baseline phenotype. |
| **Poorly infiltrated phenotype, including immune-desert states** | Low or absent pretreatment T-cell abundance within tumor tissue. Tumor type, individual immune markers, or post-treatment increases alone were insufficient for classification. |
| **Immune-excluded phenotype** | Pretreatment spatial evidence showing preferential T-cell retention in stromal, peripheral, or marginal tumor regions with limited penetration into tumor parenchyma. Bulk T-cell abundance alone was insufficient. |
| **T-cell dysfunction** | Required direct evidence of impaired effector function rather than T-cell abundance or inhibitory-receptor expression alone. |
| **T-cell exhaustion** | Required broader evidence compatible with a persistent dysfunctional program; PD-1 or another single inhibitory marker alone was insufficient. |
| **T-cell recruitment** | Used only when migration, chemotaxis, trafficking, or related recruitment evidence was reported. |
| **T-cell infiltration** | Required evidence of T-cell localization within tumor tissue. Peripheral-blood or splenic proportions alone were not considered evidence of tumor infiltration. |
| **T-cell abundance** | Used for counts, proportions, densities, or staining signals when these did not establish recruitment or function. |
| **Effector-associated readout** | Changes in IFN-γ, granzyme B, perforin, or related effector molecules were described as effector-associated readouts unless accompanied by a direct functional assay. A single effector-associated marker was not considered sufficient to demonstrate comprehensive T-cell functional restoration. |
| **Functional improvement/restoration** | Required direct functional evidence, such as cytotoxicity, tumor-cell killing, or another assay directly measuring T-cell effector activity. Multiple concordant effector-associated readouts could provide supportive evidence but were not, by themselves, interpreted as comprehensive functional restoration. |
| **ICI response/resistance** | Evaluated according to the reported response to ICI monotherapy and treatment course. Tumor type, PD-L1 expression, or combination efficacy alone was insufficient to infer response or resistance. |
| **Acquired ICI resistance** | Required evidence that resistance developed following prior ICI exposure or use of a validated acquired-resistance model. Reduced response or combination benefit alone was insufficient. |
| **Pharmacological synergy** | Reserved for studies using a predefined synergy model or formal interaction analysis. A combination outperforming the corresponding monotherapies was described as greater or additional combination activity rather than pharmacological synergy. |
| **Compound–target interaction** | Required experimental evidence supporting physical compound–target interaction beyond pathway association, such as appropriate biophysical, chemical-proteomic, or cellular target-engagement assays. |
| **Causal target/pathway requirement** | Required perturbation demonstrating that the proposed target or pathway was necessary for the relevant AS-IV-associated phenotype or treatment benefit. Compound–target interaction alone was insufficient. |

**Supplementary Table S2. Study-level methodological appraisal of the principal primary studies**

**A. Direct combinations of free AS-IV with ICIs (n = 6)**

| **Study** | **Study design and reporting** | **Immune-assay appraisal** | **Mechanistic/causal validation** | **Exposure and key methodological issues** |
| --- | --- | --- | --- | --- |
| Yang et al., 2026 (13) | RM1/C57BL/6J; n=5/group; AS-IV purity >98%. AS-IV monotherapy and anti-PD-1 combination efficacy were assessed in separate experiments rather than one matched four-arm study. Randomization: Study 1 R, Study 2 NR; blinding NR; statistics R; SSJ NR; in vitro experiments ≥3 biological replicates. | Tumor CD8 staining, chemotaxis and tumor-cell–CD8⁺ T-cell co-culture were reported; pretreatment spatial immune phenotype and intratumoral CD8 compartmentalization were not established. | SPR and CETSA supported AS-IV–PPP1R14B interaction/target engagement; genetic perturbation, degradation and ubiquitination experiments provided multi-method mechanistic support. Requirement of the CX3CL1/CD8⁺ T-cell axis for the additional anti-PD-1 benefit was not established. | PPP1R14B Kd was reported, but plasma or tumor AS-IV exposure under the efficacy regimen was not measured. Binding-site necessity and the responsible E3 ligase remain unresolved. |
| Liu et al., 2020 (14) | CT26/BALB/c; tumor-growth n=8/group and selected immune/endpoint analyses n=5; AS-IV purity NR; four treatment groups. Randomization NR; blinding NR; statistics R; SSJ NR. | Tumor macrophage and T-cell populations were assessed by flow cytometry; CD8⁺ cells were reported among CD45⁺ tumor-derived cells. Pretreatment spatial phenotype was not established. | Macrophage-associated marker changes were supported in vitro and in vivo, but no depletion or perturbation demonstrated that macrophage changes were required for combination benefit. | No plasma or tumor AS-IV PK reported. No formal pharmacological synergy analysis. |
| Wu et al., 2024 (15) | LLC/C57BL/6J; four treatment groups; animal n/group NR; AS-IV purity NR. Randomization R; blinding NR; statistics R; SSJ NR. | Immune assessment relied mainly on tissue IF for CD3, CD69, CD86 and CD206; no macrophage-lineage co-staining or direct CD8⁺ T-cell functional assay. | PI3K/Akt- and ERK-associated changes were reported, but no target-level validation or causal perturbation demonstrated pathway requirement for combination benefit. | No PK/exposure data reported. CD69 increase alone was not interpreted as comprehensive T-cell activation. |
| Wei et al., 2026 (16) | 4T1/BALB/c; 12 mice allocated to four groups, with per-group n not separately stated; AS-IV purity NR; four treatment groups. Randomization R; blinding NR; statistics R; SSJ NR. | Flow-cytometric CD3⁺CD8⁺, CD8⁺IFN-γ⁺, and CD8⁺granzyme B⁺ populations were reported, but parent populations/full gating were incompletely specified. Pretreatment spatial phenotype was not established. | H-151 supported involvement of STING-associated signaling, but direct AS-IV binding to cGAS/STING and pathway requirement for in vivo combination benefit were not established. | No PK/tumor exposure reported. The principal in vitro AS-IV concentration substantially reduced cell viability and should be interpreted alongside cytotoxicity. No formal synergy analysis. |
| Zeng et al., 2026 (17) | NPC cell models plus humanized mouse model; in vivo n=9/group; most in vitro analyses n=6; AS-IV purity NR. In vivo AS-IV route and checkpoint-antibody reporting were incomplete/inconsistent. Randomization R; blinding NR; statistics R; SSJ NR. | Human CD8⁺ T-cell co-culture included multiple effector-associated readouts; pretreatment spatial CD8 phenotype was not established. | SATB2 knockdown/overexpression and Wnt-pathway perturbation supported the SATB2/Wnt/β-catenin/PD-L1 axis; direct AS-IV–SATB2 interaction was not shown. | The identity, product information, and dose of the in vivo checkpoint antibody were incompletely or inconsistently reported and therefore require cautious interpretation. |
| Meng et al., 2021 (18) | LLC combination study directly comparing AS-IV, anti-PD-1 and combination treatment. Group size and randomization for the combination experiment were not clearly reported; a separate nude-mouse mechanistic experiment was randomized. Blinding NR; statistics R; SSJ NR. | Increased tumor-derived CD3⁺CD8⁺ proportions were reported, but the parent population and full gating strategy were not specified; pretreatment spatial phenotype was not established. | Combination activity and immune-associated changes were demonstrated, but no causal experiment established that the measured T-cell changes mediated the additional combination benefit. | No formal synergy analysis; AS-IV exposure/PK was not established in the materials examined. |

**B. Representative tumor-specific mechanistic studies without direct ICI testing (n = 9)**

| **Study** | **Study design and reporting** | **Immune/stromal-assay appraisal** | **Mechanistic/causal validation** | **Exposure and key methodological issues** |
| --- | --- | --- | --- | --- |
| Zhao et al., 2026 (75) | OSCC cell–CD8⁺ T-cell co-culture plus SCC7/C3H model; 12 mice divided into two groups; AS-IV purity >98%. Randomization R; blinding NR; statistics R; SSJ NR. | Co-culture functional readouts and tumor CD8 staining were reported, but quantitative spatial compartmentalization was not established. | MST, SPR and photoaffinity pull-down supported AS-IV–MCT1 interaction; MCT1 overexpression and mRNA-stability experiments supported functional involvement. Intact-cell target engagement, direct transport inhibition and in vivo MCT1 requirement were not established. | Binding Kd values were reported, but matched plasma/tumor AS-IV exposure was not measured. In vitro concentration reporting was internally inconsistent. |
| Ma et al., 2023 (80) | HCC cell/PBMC co-culture plus Huh-7 xenograft in BALB/c nude mice; n=5/group; experiments reported in triplicate; AS-IV purity NR. Randomization NR; blinding NR; statistics R; SSJ NR. | PBMC-mediated tumor-cell killing was assessed, but immune-cell subsets were not resolved and no tumor CD8 spatial analysis was performed. | miR-135b-5p manipulation, CNDP1 overexpression and reporter assays supported the proposed axis; no direct AS-IV target binding was shown. | No PK reported. Nude mice limit interpretation of intact T-cell-dependent antitumor immunity; SMMC-7721 has a reported identity problem. |
| Xu et al., 2018; correction 2023 (62, 63) | Lung-cancer/macrophage experiments plus LLC/C57BL/6J models; in vivo n varied by endpoint (immunological analyses n=6–7/group); AS-IV purity >98%. Randomization R; blinding NR; statistics R; SSJ NR. | Macrophage phenotyping was marker-based; CD3⁺, CD4⁺ and CD8⁺ proportions showed no significant in vivo increase. No spatial CD8 analysis. | AMPK knockdown supported pathway involvement, but no direct target binding or in vivo macrophage-dependency experiment was performed. | No PK reported. Formal correction (63) was considered together with the primary study. |
| Zhang et al., 2014 (68) | IDO-overexpressing 3LL-Luc orthotopic model; n=10/group; in vitro data reported in triplicate; AS-IV purity NR. Randomization NR; blinding NR; statistics R; SSJ NR. | Immune subsets were assessed predominantly in spleen; tumor-infiltrating Tregs, spatial CD8 distribution and direct tumor CD8 cytotoxicity were not evaluated. | IDO-overexpression context and 1-MT comparator supported pathway relevance, but direct binding, IDO enzymatic activity and kynurenine measurements were not reported. | No PK reported. CD8⁺CD28⁺ proportions were treated as CTL-associated rather than direct evidence of intratumoral functional restoration. |
| Wang et al., 2017 (50) | Patient-derived GCAF/GNF conditioned-medium study; one GCAF donor; entirely in vitro; three separate experiments; AS-IV purity NR. Randomization/blinding NA; statistics R; SSJ NR | No immune-cell or spatial CD8 assessment; study addressed CAF secretory/pro-tumor function. | miR-214 inhibition and miR-301a overexpression provided functional mechanistic support for M-CSF/TIMP2-associated effects. | PK: not applicable. Single-donor CAF source and documented BGC-823 identity problem limit generalizability; no in vivo ECM/mechanical validation. |
| Liu et al., 2023 (51) | Primary gastric CAF co-culture; n=3 replicates reported; donor replication unclear; AS-IV purity ≥98%. Randomization/blinding NA; statistics R; SSJ NR. | No immune-cell or spatial CD8 assessment. | HOXA6/ZBTB12 knockdown/overexpression, promoter-reporter and ChIP experiments supported the proposed CAF mechanism. | No in vivo exposure/PK. Tumor-cell identity concerns, unclear donor replication and absence of in vivo/immune validation limit interpretation. |
| Liang et al., 2023 (73) | AOM/DSS tumorigenesis model; primary in vivo outcome n=6/group; AS-IV purity ≥98%. Randomization R (SPSS random-number method); blinding NR; statistics R; SSJ NR. | No defined tumor CD8/Treg/TAM functional analysis or spatial immune assessment. | SPR and GW9662 intervention supported PPARγ-related pharmacology, but the study did not establish checkpoint-relevant immune causality in established tumors. | No tumor/plasma AS-IV PK reported. Model is inflammation-associated tumorigenesis rather than a direct ICI-response model. |
| Min et al., 2022 (64) | Huh-7/THP-1-derived macrophage co-culture plus Huh-7/macrophage co-implantation in BALB/c nude mice; n=10/group; experiments performed at least three times; AS-IV purity NR. Randomization R; blinding NR; statistics R; SSJ NR. | Macrophage-associated markers were assessed; CD8 infiltration and spatial compartmentalization were not reported. | TLR4 siRNA and pathway readouts supported TLR4/NF-κB/STAT3 involvement, but in vivo macrophage dependency was not established. | No PK reported. Immunodeficient host and direct tumor-cell effects complicate macrophage-specific attribution; some siRNA and SD/SEM reporting was inconsistent. |
| Zhou et al., 2024 (65) | MC38/RAW264.7 conditioned-medium studies plus intrasplenic MC38 liver-metastasis model; n=3/group in vivo; AS-IV purity NR. Randomization R; blinding NR; statistics R; SSJ NR. | Macrophage-associated proportions were reported; CD8 infiltration and tissue compartmentalization were not assessed. | EV characterization, GW4869 comparison and EV add-back experiments supported an EV-related mechanism, but in vivo dependence on EV suppression or macrophage changes was not established. | No PK reported. Small animal groups and dosing initiated before tumor inoculation limit therapeutic inference. |

**C. ICI combination studies using AS-IV-containing formulations or delivery systems (n = 5)**

| **Study** | **Study design and reporting** | **Immune/stromal-assay appraisal** | **Mechanistic/causal validation** | **Exposure and key methodological issues** |
| --- | --- | --- | --- | --- |
| Guo et al., 2024 (58) | Orthotopic HCC nanoplatform study; tumor endpoints n=5/group, survival n=8/group, selected immune analyses n=3; AS-IV purity NR. Randomization R; blinding NR; statistics R; SSJ NR. | TIL-associated and vascular/hypoxia measures were reported, but baseline spatial phenotype and quantitative CD8 compartmentalization were not established. | Multiple vascular and immune readouts supported platform-level activity, but AS-IV-specific causality was not isolated. | No direct AS-IV PK; carrier distribution is not equivalent to AS-IV exposure. Multicomponent platform lacked matched single-component controls; reported H22-Luc/C57BL/6 compatibility requires clarification. |
| Wei et al., 2025 (77) | Orthotopic 4T1-Luc model; tumor response n=6/group, survival n=8/group, PK n=6; AS-IV purity >98%. Randomization R; blinding NR; statistics R; SSJ NR. | CAF/stromal measures and CD8⁺ TIL functional readouts were included; no quantitative spatial CD8 compartmentalization. | Mechanistic evidence supported formulation-associated CAF/TIL effects, but individual contributions of AS-IV, oxymatrine and liposomal delivery could not be separated. | Quantitative plasma and tumor AS-IV AUC and Cmax were reported. Formulation-associated exposure was measured, but the individual contributions of AS-IV, oxymatrine, and liposomal delivery could not be separated. |
| Liu et al., 2025 (72) | Pulmonary LLC-Luc model; combination experiment involved 12 mice divided into four groups; AS-IV purity NR. Randomization R for in vivo studies; blinding NR; statistics R; SSJ NR. | Tissue staining and flow cytometry were used, but quantitative CD8 spatial distribution was not established. | Transcriptomic/pathway-associated evidence and inhibitor comparisons were reported, but pathway necessity for combination benefit was not established. | A 24-h lung-tissue AS-IV concentration was reported, but no complete concentration–time profile was reported. No single-component control; acquired anti-PD-1 resistance was not established before treatment. |
| Guo et al., 2023 (78) | Orthotopic HCC multicomponent nanoplatform; efficacy/survival n=8/group, selected flow assays n=5, Seahorse assays n=3; AS-IV purity NR. Randomization R; blinding NR; statistics R; SSJ NR. | Post-treatment TIL flow and CD8 staining were reported without tumor-nest/stromal/margin quantification. | Separate stromal and TIL-metabolic experiments supported platform-level mechanisms, but AS-IV-specific immune causality was not established. | No AS-IV PK. No matched in vivo single-drug or single-drug-loaded controls; carrier itself altered TIL metabolism; reported H22-Luc/C57BL/6 pairing requires clarification. |
| Kong et al., 2024 (79) | Orthotopic MB-49/C57BL/6 bladder model; efficacy/survival n=5/group, IHC/flow n=3; purity NR; randomization/blinding NR; statistics R; SSJ NR; ARRIVE reported. | Post-treatment CD8 IHC and flow-cytometric immune changes were reported without spatial compartmentalization; flow reporting was incomplete. | STAT3/NF-κB-associated changes were reported, but pathway necessity was not established. | No quantitative AS-IV PK. No antibody-only or antibody-decorated empty-micelle control; conjugation and dosing sequence differed simultaneously, and dose normalization was unclear. |

**Abbreviations:** R, reported; NR, not reported in the materials examined; NA, not applicable; SSJ, sample-size justification; IF, immunofluorescence; PK, pharmacokinetics; SPR, surface plasmon resonance; MST, microscale thermophoresis; CETSA, cellular thermal shift assay; Kd, dissociation constant; EV, extracellular vesicle; CAF, cancer-associated fibroblast; GCAF, gastric cancer-associated fibroblast; GNF, gastric normal fibroblast; TIL, tumor-infiltrating lymphocyte; PBMC, peripheral blood mononuclear cell; HCC, hepatocellular carcinoma; OSCC, oral squamous cell carcinoma; NPC, nasopharyngeal carcinoma; CTL, cytotoxic T lymphocyte; AUC, area under the concentration–time curve; Cmax, maximum concentration; HPLC, high-performance liquid chromatography; ARRIVE, Animal Research: Reporting of In Vivo Experiments. Categories A–C indicate evidence type rather than methodological quality. No aggregate quality score was assigned.
